# Regulation at a distance of biomolecular interactions using a DNA origami nanoactuator

**DOI:** 10.1038/ncomms10935

**Published:** 2016-03-18

**Authors:** Yonggang Ke, Travis Meyer, William M. Shih, Gaetan Bellot

**Affiliations:** 1 Wallace H. Coulter Department of Biomedical Engineering, Georgia Institute of Technology and Emory University, Atlanta, Georgia 30322, USA; 2 Wyss Institute for Biologically Inspired Engineering, Harvard University, Boston, Massachusetts 02115, USA; 3 Department of Cancer Biology, Dana-Farber Cancer Institute, Harvard Medical School, Boston, Massachusetts 02115, USA; 4 Department of Biological Chemistry and Molecular Pharmacology, Harvard Medical School, Boston, Massachusetts 02115, USA; 5 Department of Neuroscience, Institut de Génomique Fonctionnelle, Centre National de la Recherche Scientifique, CNRS Unité Mixte de Recherche UMR 5203, Institut National de la Santé et de la Recherche Médicale, INSERM U1191, 141 rue de la Cardonille, F-34000 Montpellier, France; 6 Université de Montpellier, F-34000 Montpellier, France

## Abstract

The creation of nanometre-sized structures that exhibit controllable motions and functions is a critical step towards building nanomachines. Recent developments in the field of DNA nanotechnology have begun to address these goals, demonstrating complex static or dynamic nanostructures made of DNA. Here we have designed and constructed a rhombus-shaped DNA origami ‘nanoactuator' that uses mechanical linkages to copy distance changes induced on one half (‘the driver') to be propagated to the other half (‘the mirror'). By combining this nanoactuator with split enhanced green fluorescent protein (eGFP), we have constructed a DNA–protein hybrid nanostructure that demonstrates tunable fluorescent behaviours via long-range allosteric regulation. In addition, the nanoactuator can be used as a sensor that responds to specific stimuli, including changes in buffer composition and the presence of restriction enzymes or specific nucleic acids.

## Abstract (web summary)

The construction of nano-machines requires building nano-scale structures with controllable functions. Here the authors use DNA origami to construct an allosteric actuator which can act as signal propagator and an environmental sensor.

## Graphical abstract

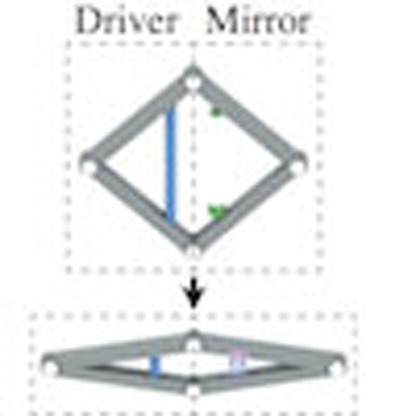

Biomolecules that respond to chemical and physical stimuli and go through conformational changes are crucial in cellular functions, such as transmembrane signalling, intracellular transport and cell migration and division. The engineering of rationally designed artificial nanodevices, which mimic those conformational changes can facilitate understanding of the molecular mechanisms behind these biomolecular machines. In addition, such man-made nanodevices may find applications in areas such as biosensing, biophysics, on-demand drug delivery, photonics and energy harvesting.

DNA origami self-assembly is one of the most promising routes towards building such artificial biomolecules. The last decade has seen the construction of a large library of complex nanoscale objects built using this approach—a long scaffold strand derived from an M13 viral genome was folded with hundreds of short synthetic staple strands into custom-designed, fully addressable, two- or three-dimensional structures^1^,^2^,^3^,^4^,^5^,^6^,^7^,^8^,^9^. Furthermore, these DNA origami nanostructures can be used as molecular pegboards with nanometre resolution and have been widely employed for the assembly of heteroelements such as proteins^10^,^11^,^12^, viruses^13^ and nanoparticles^14^,^15^,^16^. Most DNA origami nanostructures constructed to date have primarily been static objects, although a number of dynamic DNA origami structures also have been demonstrated with kinematic joints^17^, shape complementarity^18^ and compliant mechanism designs^19^. Using these principles, several reconfigurable DNA origami devices have been developed, such as nanoreactors^20^, Brownian ratchets^21^, nanopores^22^, plasmonic nanostructures^23^, reconfiguring structures^24^,^25^, nanojoints^26^ and nanocontainers for controlled payload release^3^,^27^,^28^,^29^,^30^. Of particular note, Gu *et al*.^31^ and Liu *et al*.^32^ demonstrated the use of DNA ‘nano-scissors', built from double cross-over tiles, which can actuate movement between arms to report on work done by the DNA-binding MutS protein or control distance between enzyme/cofactor pairs, respectively^31^,^32^. However, these devices only show movement over small distances, which limits the functionality of the design. Kuzuya *et al*.^33^ built a DNA origami ‘nano-plier', which displays large-scale reconfiguration on binding to a target analyte, although the actuation is limited to a two-step (open/close) process. A useful capability for these dynamic structures would be an allosteric activation mechanism that propagates over a long distance so as not to affect substrate availability or reactivity, a trait not realized in previous works.

Here we demonstrate the ability to control a dynamic DNA origami with long-range allosteric activation properties, wherein the binding of an effector molecule controls its global shape. The origami device, which we call a ‘nanoactuator' (NA), consists of four stiff arms connected into a rhombus shape such that the angles between opposing joints must be mirrored. This design enables precise distance changes induced in one half of the rhombus to be propagated quantitatively to the other half of the rhombus, allowing for large-scale movements in a multi-step process. We expect our work on the development of a nanometre-sized device with precisely engineered motion will broaden the scope of DNA nanostructures in single-molecule biophysics and biosensing applications.

## Results

### Design and characterization of the DNA origami device

**Figure 1:**
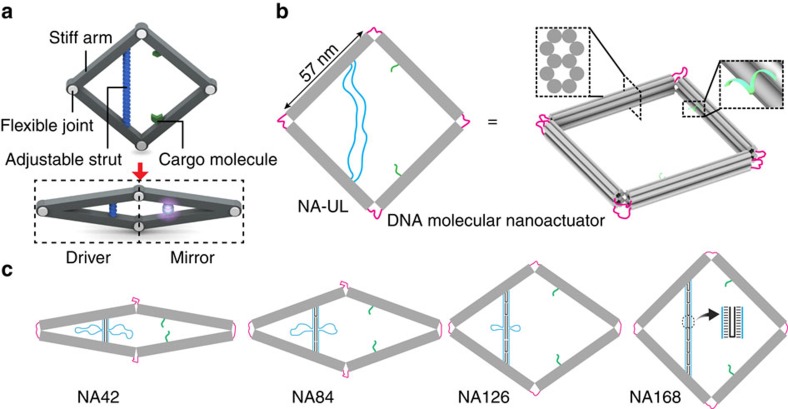
Design of the tunable DNA origami nanodevice. (**a**) Three-dimensional models of the nanodevice that controls the interaction between two cargo molecules by adjusting the distance between them. (**b**) The four-arm device has two capturing strands (green) for cargo molecule attachment. For controlling its conformation, the devices also have two single-stranded segments of scaffold connecting the two left arms (blue) and two short single-stranded segments of scaffold (pink; it is noteworthy that only one is shown) connecting each corner. Without locking strands, the device is flexible and can adopt different conformations constrained within the plane defined by the four arms. (**c**) Using ‘strut-locking' strands, nanoactuator conformation could precisely tune from closed stage to open stage by adjusting the length of the connecting struts. The nanoactuator was adjusted to form NA42, NA84, NA126 and NA168 using strut-locking strands (black).

A DNA–protein hybrid molecule with tunable fluorescence properties was created by attaching split enhanced green fluorescent protein (eGFP) to the DNA nanodevice (Fig. 1a). In addition, we also show that this versatile DNA nanodevice can function as a nanosensor in response to different types of stimuli, including changes in buffer composition, enzymatic activity and nucleic acid hybridization.

The NA was created using the DNA origami method: an M13 scaffold strand and hundreds of short DNA strands self-assemble to form the final nanostructure. This nanodevice contains four stiff, ten helix-bundle (10HB) arms that are connected by flexible joints (Fig. 1b), consisting of single-stranded portions of the scaffold strand. The arms were designed with a 10HB structure to resist deformation under bending, shearing or torsional stresse—the upper bound of the persistence length of the 10HB arm was calculated to be 6.8 μm, using equations outlined in Castro *et al*.^34^. To limit out-of-plane movement, each pair of neighbouring arms was connected by two flexible hinges that restrict movement of the nanodevice within a two-dimensional plane (Fig. 1b, pink). To control the conformation of the device, unpaired single-stranded scaffold regions were incorporated into the design: (1) two segments of 168-NT (nucleotide) scaffold connect the two left arms of the devices (Fig. 1b, blue) and (2) each corner of the device contains two short segments of 42-NT single-stranded scaffold DNA (Fig. 1b). Each of the two right arms contains short single-strand DNA extensions that allow for immobilization of cargo molecules (Fig. 1b, green). Both the target anchoring strands on the right arm and 168NT scaffold on the left arm are located 44 nm from the right and left vertices, respectively, such that the distance between the upper and lower arms at these points is directly mirrored across the device. More details of the NA design are shown in Supplementary Figs 1 and 2.

We designed two different mechanisms to control the movements of the NA (Fig. 1c). One mechanism controls the device's conformation by adding extra ‘strut-locking' strands to form two rigid DNA double helices we call the ‘connecting strut' (Fig. 1c, black and blue strands). The connecting strut can be adjusted to a custom-designed length ≤168 bp; in this work, we demonstrated the use of connecting struts with four different lengths: 42, 84, 126 and 168 bp. Correspondingly, we have named the NA assembled with those strut-locking strands NA42, NA84, NA126 and NA168, respectively.

**Figure 2:**
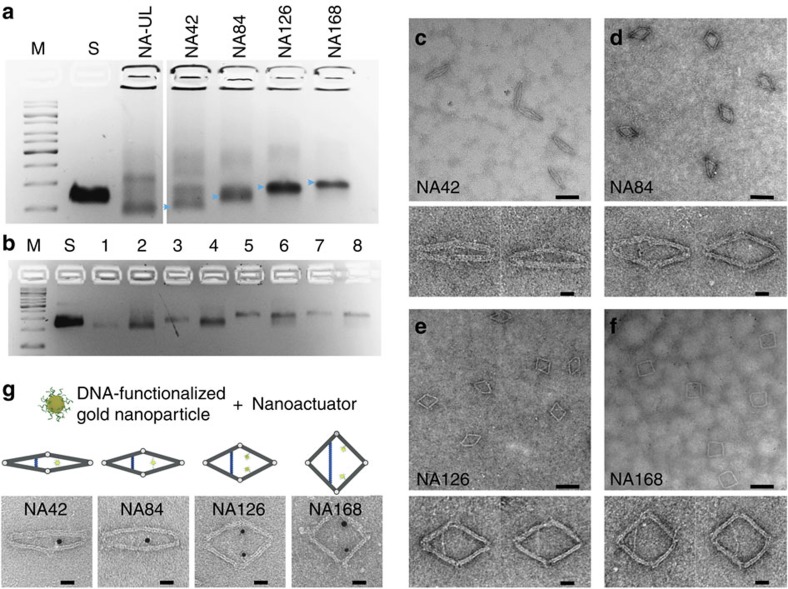
Gel electrophoresis and TEM characterization of the device. (**a**,**b**) Agarose gel electrophoresis results. (**a**) Lane M, 1 kb DNA ladder. Lane S, p7560 scaffold. Lane NA-UL, nanoactuator assembled without locking strands. Lane NA42 to NA168 nanoactuator assembled with 42, 84, 126 and 168 bp locking strands. Arrows detonate the main product bands. (**b**) Lane M, 1 kb DNA ladder. Lane S, the p7560 scaffold. Lane 1, purified NA42. Lane 2, purified NA-UL mixed with 42 bp locking strands. Lane 3, purified NA84. Lane 4, purified NA-UL mixed with 84 bp locking strands. Lane 5, purified NA126. Lane 6, purified NA-UL mixed with 126 bp locking strands. Lane 7, purified NA168. Lane 8, purified NA-UL mixed with 168 bp locking strands. (**c**–**f**) TEM image of purified products from agarose gel in **a**. (**c**) TEM images of purified NA42. (**d**) TEM images of purified NA84. (**e**) TEM images of purified NA126. (**f**) TEM images of purified NA168. (**g**) Three-dimensional models (top) and TEM images (bottom) showing the device capturing gold nanoparticles. Purified NA42, NA84, NA126 and NA168 were mixed with 5 nm gold nanoparticles functionalized with thiol-modified single-stranded DNA whose sequence was complementary to the capturing strands on devices. Each NA42 or NA84 captured with one gold nanoparticle, whereas each NA126 or NA168 captured two gold nanoparticles. Scale bars, (**c**–**f**) 100 nm (top), 20 nm (bottom); (**g**) 20 nm.

The NA was assembled by mixing scaffold and staple strands together at a 1:10 molar ratio in 5 mM Tris, 1 mM EDTA pH 8.0, supplemented with 14 mM MgCl_2_. The solution was then subjected to an 18-h thermal annealing process. We first tested a one-step assembly process and analysed the results by native agarose gel electrophoresis (Fig. 2a). In the first experiment, a total of five samples were assembled: the NA without locking strands, NA42, NA84, NA126 and NA168. Each sample produced a distinct product band corresponding to the expected mobility. The NA without any locking strands showed the fastest mobility (Fig. 2b, lane 1), because the flexible device could more easily adapt its conformation to the path of least resistance when migrating through the pores of the agarose gel. NA42, NA84, NA126 and NA168 exhibited decreasing mobility on the gel (Fig. 2b, lane 4 to lane 7), owing to their gradually more open conformation. The smearing associated with NA-UL is probably a representation of the heterogeneity of conformations the structure can exist in when in an unlocked state, whereas the lower mobility bands in NA-UL and NA42 correspond to dimers formed via base stacking interactions (Supplementary Fig. 3). Controlling the conformation of the NA could also be achieved in a two-step reaction process (Fig. 2b). We first assembled the NA without any locking strands and then purified it from an agarose gel following electrophoresis. Next, the 42-, 64-, 126- and 168-bp locking strands (1 pmol per strand) were added to 10 μl (with an estimated concentration of ∼5 nM) of the purified flexible device and were left to react for 2 h at room temperature before a second round of agarose gel electrophoresis. Each reaction produced a distinctive major band (Fig. 2b, lanes 2, 4, 6 and 8) that showed similar mobility as the corresponding device assembled in the one-step reaction (Fig. 2b, lanes 1, 3, 5 and 7).

The one-step-assembled NA42, NA84, NA126 and NA168 were then characterized by negative-stained transmission electron microscopy (TEM) imaging (Fig. 2c–f). Well-formed NAs were clearly visible in the images. Most structures showed the designed morphologies. More TEM images of the NAs and detailed analysis of their morphologies are included in Supplementary Figs 4–9.

To demonstrate that cargo molecules can be attached to the two capturing strands, we added 5 nm gold nanoparticles to the NA. The 5-nm gold nanoparticles were first functionalized with thiolated single-stranded DNA with a 5′-thiol-TTTTTTTTTT-3′ sequence. The poly-T DNA-covered gold nanoparticles were then mixed at room temperature with the NA devices containing 5′-staple sequence-AAAAAAAAAA-3′ capturing strands. TEM images showed the gold nanoparticles were successfully attached to the device (Fig. 2g). Owing to the close proximity of the two capturing strands, NA42 or NA84 typically captured only one gold nanoparticle. In contrast, NA126 or NA168 typically captured two gold nanoparticles at the designed binding sites.

### Construction of an allosteric biomolecular complex

**Figure 3:**
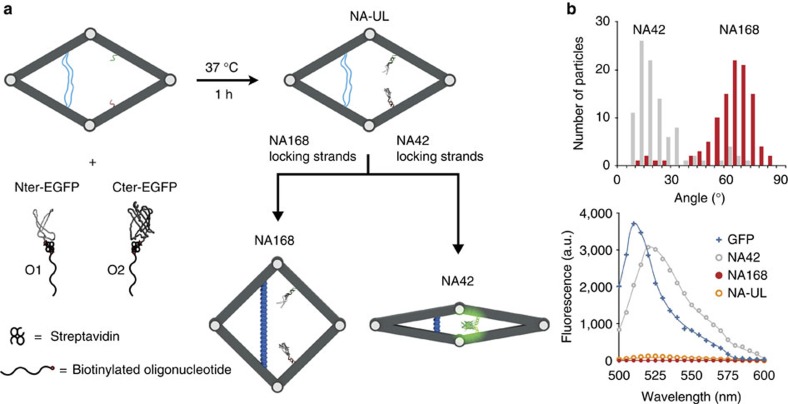
Schematic representation of eGFP complementation triggered by allosteric regulation in the DNA nanodevice. (**a**) Split eGFP constructs and assembly. The eGFP fluorescent protein is split in two non-fluorescent fragments, a large N-terminal eGFP fragment and a small C-terminal eGFP fragment. Each protein fragment is linked to an oligonucleotide via biotin:streptavidin interactions. Oligonucleotides O1 and O2 are linked to N-terminal eGFP fragment and C-terminal eGFP fragment, respectively. The two nucleoproteins associate with the DNA nanodevice by sequence-specific duplex DNA formation at 37 °C for 1 h. Purified split eGFP nanodevice conjugates are stabilized in the open state by adding NA168 strut-locking strands or in closed state by adding NA42 strut-locking strands set. (**b**) TEM and fluorescence intensity analysis of the open (in red) and closed (in grey) device. Shown in blue are the fluorescence spectra of intact eGFP and in orange are the fluorescence spectra of unlocked state device. The software ImageJ was used to manually measure the angles of each structure from TEM images.

Biomolecules often exhibit allosteric regulation of their conformational changes, such that the initial response to stimuli is transferred across relatively long distances within the molecule. The enzyme pyruvate kinase and oxygen-carrying protein haemoglobin both exhibit allosteric regulation of their functionality^35^,^36^. We modified the NA design to behave as a ‘nanoswitch' that can mimic this allosteric regulation, dynamically varying the proximity between two guest biomolecules so as to achieve cycles of active and inactive states. We selected the two halves of eGFP as a test case (Fig. 3a). We first separated eGFP in two non-fluorescent fragments, as described previously: a larger amino-terminal eGFP fragment (158 amino acids) that contains a quenched chromophore and a smaller carboxy-terminal eGFP fragment (81 amino acids)^37^. N-terminal and C-terminal eGFP fragments with an additional cysteine residue added to the C- and N termini, respectively, were cloned into the pTWIN-1 vector, to yield the C-terminal fusions to the SspDNAB intein. Both fragments were then expressed and purified independently (Supplementary Fig. 10). The two non-fluorescent fragments were conjugated to two DNA strands, which then bound to two complementary capturing DNA strands incorporated on the nanoswitch. The device's NA-UL to NA42 (‘unlocked' to ‘closed') conformational change will bring the two split eGFP fragments to close proximity, resulting in a restoration of strong fluorescence. On the other hand, NA-UL to NA168 (‘unlocked' to ‘open') conformational change of the device will lead to complete loss of fluorescence. Based on the angular distributions of both the NA42 and NA168 states, the estimated distance between the two eGFP fragment anchoring points in these confirmations is 15 and 60 nm, respectively.

We used a streptavidin-biotin bridging method to attach the split eGFP fragments to DNA strands. (Fig. 3a and Supplementary Fig. 11). The two purified fragments were first biotinylated with the sulfhydryl-reactive reagent biotin-N-[6-(biotinamido)hexyl]-3′-(2′-pyridyldithio)propionamide (HPDP) via disulfide bond formation to their terminal cysteine residues. The N-terminal and C-terminal biotinylated fragments were then coupled with two distinct biotinylated oligonucleotides (5′-TCGGTCACAGTACAACCGTTATCTACATA-3′-Biotin and 5′-Biotin-TATGTAGATTTTCCCATTGCTCTTAATTGTT-3′, respectively) at a 1:1 ratio via dual biotin binding to streptavidin, which acts as a linker protein. The two DNA sequences contain two nine-base complementary regions that help to drive the complementation of the two eGFP fragments by sequence-specific duplex DNA formation, as described previously^37^. Two complementary anchor strands were attached to the two opposite right arms on an unlocked device. Each anchor strand is designed to be 44 nm away from the right corner (Fig. 3a). The unlocked DNA origami was separated from excess staple strands by agarose gel electrophoresis and purified via electroelution^38^. The nanodevice was then subsequently incubated for 1 h at 37 °C with two DNA-functionalized split eGFPs at a molar ratio of 1:5:5. The complete protein/DNA origami nanoswitches were then purified by rate-zonal centrifugation^39^ and buffer-exchanged with 10 mM PBS buffer (pH 7.4, with 160 mM NaCl and 10 mM MgCl_2)_. This purification procedure produced highly pure protein/DNA origami hybrid nanoswitches, confirmed by agarose gel electrophoresis (Supplementary Fig. 12). The purified protein/DNA origami samples were concentrated to ∼250 nM before further use.

We then tested the hybrid nanoswitches at its open (NA168) and closed (NA42) states. Purified nanoswitches samples (50 μl at ∼250 nM) were mixed with a fivefold molar excess of 168 or 42 bp strut-locking strands. After 2-h incubation at room temperature, we first characterized the nanoswitches via TEM by measuring the angles of individual structures in TEM images. The majority of open structures and closed structures exhibited angles of 60°–70° and 10°–20°, respectively. Fluorescence was then used to monitor the hybrid nanoswitch conformational change and interaction between the two split eGFP fragments. At the open position, split eGFP fragments were weakly fluorescent, as expected when the two molecules were held apart (Fig. 3b). In comparison, split eGFP fragments in the unlocked state showed only slightly stronger fluorescence signal (Fig. 3b). However, for the closed hybrid nanoswitch, a significant increase in fluorescence with maximal emission at 524 nm was observed, consistent with our design efficiently bringing together the GFP fragments. A continuous increase in fluorescence was observed and stabilized at a final steady state after 280 s, suggesting re-assembly of complete eGFP had reached equilibrium (Supplementary Fig. 13).

### Spring-loaded nanosensors

**Figure 4:**
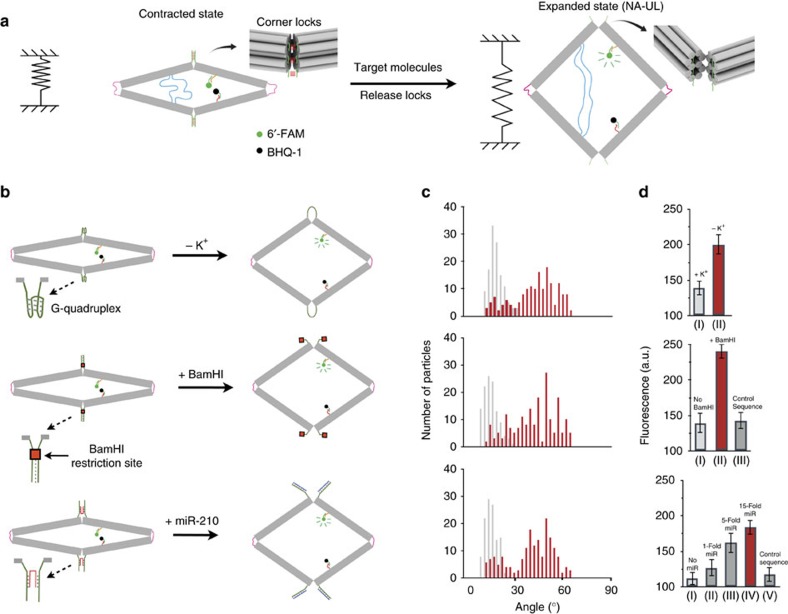
Design and analysis of the DNA origami nanosensors. (**a**) Schematic view of the closed and opened state of the device loaded with a fluorophore (6-FAM) and a quencher (BHQ-1). In the closed state, fluorophore (6-FAM) and quencher (BHQ-1) are within a Forster radius distance and there is low emission. As the device is relaxed in the presence of a target molecule, the fluorophore and the quencher are moved further away and light is emitted. (**b**) Schematic illustration of the working principles of the nanosensor-based assay. The lock system in the top and bottom corners was design to recognized three different stimuli: (1) a buffer-sensitive activation paradigm with a G-quadruplex as the sensing element; (2) an enzyme-sensitive activation paradigm containing a specific palindromic sequence of nucleotides, which is recognized by the enzyme BamHI; (3) a nucleic acid-sensitive activation paradigm, using hybridization of the microRNA miR-210, which is a sensor for hypoxic stress during tumorigenesis. (**c**) Histogram distributions of the conformational analysis from TEM experiments, grey is the pre-stressed nanoswitches and (**d**) measurements of the fluorescence intensity (arbitrary units) emitted by a 50 nM solution of origami of each system. For buffer-sensitive activation: (I) in the closed state and (II) in presence of 100 mM KCl. For enzyme-sensitive activation: (I) in the closed state, (II) in presence of five units of BamHI after 10 min incubation, (III) after 30 min of incubation and (IV) negative control with locking strands lacking BamHI site. For small molecule-sensitive activation: (I) in the closed state, (II) in the presence of 1 equivalent of miR-210 to each locking strand, (III) in the presence of 5 equivalents of miR-210, (IV) in the presence of 15 equivalents of miR-210 and (V) after the addition of a control competitor that was not complementary to the target. Each bar is the average of three independent measurements.

We further evolved the device to a ‘spring-loaded' DNA origami actuator that selectively responds to several different external stimuli. The device acts as pre-compressed springs that keep its closed conformation via different locking mechanisms. In presence of external triggers, the locks dissociate and the device reconfigures to the NA-UL state, which is mostly open due to electrostatic repulsion of the DNA origami arms (Fig. 4a). To generate the initial closed state, we incorporated three new specifically designed staple strands at the top and bottom corners of the device during the folding of the DNA origami. Details of each design are shown in Fig. 4b and Supplementary Fig. 14. We demonstrated three sensors that responded to changes in buffer composition, restriction enzymes and nucleic acids, respectively. The closed-to-open conformational change was characterized by either single-molecule imaging using TEM or by monitoring the fluorescence signal change of 6-FAM (6-carboxyfluorescein)/BHQ-1 dye/quencher pair. Transition to an open configuration increases the distance between dye and quencher, leading to recovery of the fluorescent signal).

*Buffer-sensitive activation*. The device was designed with G-quadruplex DNA structures as sensing elements for selective potassium (K^+^) detection^40^. The potassium-triggered device consists of three top-corner locking strands and three bottom-corner locking strands. Each locking strand has a 34-base single-stranded overhang with sequence 5′-TACAGGGGTGTGGGGACAGGGGTGTGGGGTACAT-3′, which forms an antiparallel G-quadruplex motif in the presence of K^+^. The working principle is depicted in Fig. 4b and Supplementary Fig. 15. TEM analysis of the purified pre-stressed device in the presence of 100 mM KCl shows a characteristic angle distribution ranging from 10° to 25° with an average angle of 20° corresponding to the closed state (Fig. 4c and Supplementary Fig. 16). Buffer exchange of the solution (10 mM Tris pH 8.0, 10 mM MgCl_2_) that selectively removes the K+ from the system yielded a significant increase in the fluorescence of 6-FAM (44% enhancement; Fig. 4d), corresponding to the device's open conformation. The open state was further confirmed through TEM analysis, which showed an angle distribution from 30° to 60° (Fig. 4c and Supplementary Fig. 17).

*Enzyme-sensitive activation*. The enzyme-sensitive origami device explored the feasibility of driving the configuration to the open state via an enzyme-catalysed hydrolysis process. The enzyme-sensitive origami device is pre-stressed via DNA locking strands that contained a specific sequence (5′-TGCGCGCGGATCCGCGCAAGCGCGCGCCTAGG-3′), whose palindromic region allowed the locking strands to form dimers that were recognizable by the restriction enzyme BamHI (Fig. 4b and Supplementary Fig. 15). Agarose-gel-purified pre-stressed devices showed an average angle distribution of 20° in TEM images (Fig. 4c and Supplementary Fig. 18). After 10- and 30-min incubation with BamHI, the pre-stressed devices exhibited 41% and 68% enhancement of 6-FAM emission, respectively (Fig. 4d). The final open state of the device was further confirmed by TEM images, which showed an increased average angle of 50° (Fig. 4c and Supplementary Fig. 19). For comparison, a negative control sample was assembled by incorporation of locking strands without the BamHI recognition site. The fluorescence emission of the device was not affected by the presence of BamHI (Fig. 4d).

*Nucleic-acid-sensitive activation*. The device was also modified to demonstrate selectively detection of human microRNAs. Single-stranded DNA extensions that contain complementary sequence to miR-210 (5′-CTGTGCGTGTGACAGCGGCTGA-3′, a marker for hypoxic stress during tumorigenesis^41^) were added to the top and bottom corners. A short strand was added to lock the device (Fig. 4b bottom and Supplementary Fig. 15). We tested a series of lengths of locking strands to achieve optimal miR-210 detection efficiency. We found that shorter locking gave better miR-210 detection sensitivity, but at the expense of reduced assembly yield of pre-stressed closed device. We finally chose a 20-base lock strand (10-bases on each overhang) for subsequent experiments, because it offered high sensitivity for miR-210 binding and >70% yield of the closed device before purification (Supplementary Fig. 13). A 12-base single-stranded toehold was used to trigger the strand displacement in the presence of miR-210.

The closed device was gel purified and buffer exchanged into PBS. TEM images showed that most of the devices had angles between 10° and 20° (Fig. 4c and Supplementary Fig. 20). Selective detection of miR-210 was carried out by 2-h incubation at 37 °C of 100 nM device and miR-210 at three different concentrations: 1, 5 and 15 equivalents to the locking strand. Thirteen per cent and 58% enhancement of 6-FAM emission were observed in the presence of 1 to 15 equivalents miR-210, respectively (Fig. 4d). Complete hybridization of the miR-210 target to the device is observed when the microRNA is present at 15 equivalents to each locking strand. TEM images showed an angle distribution from 35° to 60°, further demonstrating successful opening of the device (Fig. 4c and Supplementary Fig. 21). For comparison, adding a random 22-mer sequence (5′-TATGTAGATGATTGCTAGATC-3′) in large excess (50 equivalents to each locking strand) did not show any enhancement of 6-FAM emission after a 2-h incubation at 37 °C (Fig. 4d).

All nucleic acid sequences used for the nanosensors are shown in Supplementary Table 1.

## Discussion

In summary, we have successfully constructed an artificial DNA–protein hybrid nanoswitch whose fluorescence signal could be turned on and off by tuning the DNA origami scaffolding nanostructure. The molecule's conformation was precisely controlled by altering the length of a ‘connecting strut'. The same mechanism can potentially be applied to other proteins, small molecules and nanoparticles for constructing DNA-controlled artificial nanodevices with tunable enzymatic, photonic or plasmonic functions.

In addition, the nanodevice can serve as a general platform for studying weak molecular interactions at the single-molecule level (for instance, weak protein–protein interactions). Single-molecule experiments are generally limited to strongly interacting pairs, because the short binding time between weak pairs often make it impossible to monitor. In addition, single-molecule methods typically require low concentrations of molecules, which further reduce the frequency of weak molecular interactions. Our device provides a potential solution for observing these weak interactions; by increasing the local concentration of molecules and by providing rationally designed spatial confinement, we may enhance the weak interactions to enable monitoring such events at the single-molecule level.

We also demonstrated another open/closed mechanism using only ‘corner-locking' strands. This novel mechanism allowed us to build a group of sensors that respond to a wide range of stimuli, including changes in buffer composition, restriction endonuclease enzymatic activity and RNA–DNA hybridization. In these sensors, the sequences of the corner-locking strands were designed independently without regard to the underlying DNA origami structures that carried the fluorescence reporters. Therefore, these sequences can be modified to enable the DNA origami nanodevice, to sense other types of stimuli ranging from small molecules to large protein complexes. For instance, implementing aptamer sequences can potentially allow the device to detect specific proteins.

Finally, the nanodevice could be adapted to serve as a quantitative platform for a low-cost electrophoretic mobility shift assay similar to what was recently reported by Koussa *et al*.^42^ The allosteric mechanism and the large size of the origami devices are two of the advantages for stimuli detection using this technique. Alternatively, the drastic conformational change of the device in the presence of specific targets can be visually detected at the single-molecule level through atomic force microscopy or electron microscopy. For fluorescence-based detection, the three-dimensional shape and the chemical addressability of the origami device allows for the incorporation of several dye/quencher pairs along the arms of the device, thereby making readout of the nanoswitches more sensitive.

## Methods

### Materials

All staple strands were purchased from Bioneer Inc., purified by reverse-phase cartridge. Sequences of dye labelled strands are as follows: 5′-FAM-CGGTTGTACTGTGACCGA-3′ and 5′-BHQ1-GAATCGGTCACAG-3′. Biotin-HPDP was purchased from Pierce. The p7560 scaffold strand was produced from M13 phage replication in *Escherichia coli*. The inoculation phage is produced in two steps (preinoculation and inoculation), to ensure sufficient quality and quantity^2^. The BamHI enzyme was purchased from New England Biolabs.

### Design and assembly of DNA origami structures

The DNA origami nanostructures were designed using the honeycomb-lattice version of the caDNAno software. Assembly of the 10HB device was accomplished in a one-pot reaction by mixing scaffold strands derived from M13 bacteriophage (p7560) at 10 nM with 100 nM of each oligonucleotide staple strand in a folding buffer containing 5 mM Tris, 1 mM EDTA pH 8, supplemented with 14 mM MgCl_2_. The mixture was subjected to a thermal-annealing ramp: 80 °C for 5 min, −1 °C per cycle for 14 additional cycles, 65 °C for 20 min, −1 °C per cycle for 35 additional cycles.

### Nanosensor device

Assembly of each nanosensor device was accomplished in a similar manner as the nanoacuator in the presence of six equivalents of dye-labelled staples and six equivalents of locking strands.

### Agarose gel analysis

Annealed DNA structures were purified by 2% agarose gel electrophoresis (0.5 × TBE (45 mM Tris-borate, 1 mM EDTA pH 8.3), 11 mM MgCl_2_ and 0.5 mg ml^−1^ ethidium bromide) with a Thermo Scientific Owl B2 EasyCast Mini Gel System apparatus in an ice-water bath. The DNA structure samples were loaded into the agarose gel and allowed to migrate for 3 h (running buffer: 0.5 × TBE, 11 mM MgCl_2_ running voltage: 2.85 V cm^−1^). The migrating bands corresponding to the correctly folded structures were then visualized with ultraviolet light and cut out from the gel. Excised bands were crushed and transferred into DNA gel extraction spin column (Bio-Rad, catalogue number: 732–6166). The DNA structure solution was recovered by centrifugation of the loaded column for 10 min at 16,000 *g*.

### Nanosensor activation

For buffer-sensitive activation, the annealed device was first buffer exchanged using a Slide-A-Lyzer MINI Dialysis Unit, (2,000 molecular weight cut off) from Thermo Scientific. The dialysis unit was floated in 250 ml of potassium buffer (10 mM Tris pH 8.0, 10 mM MgCl_2_ and 100 mM of KCl), to stabilize the G-quadruplex DNA captured inside the device. Buffer was replaced three times over 2 h at room temperature for small samples (for example, 250 μl). After dialysis, the pre-stressed device was recovered from the dialysis unit and separated by the rate-zonal centrifugation method^35^. The running buffer was supplemented with 100 mM KCl. The 45% fractions containing the closed device were combined and buffer exchanged, to remove the glycerol (10 mM Tris pH 8.0, 10 mM MgCl_2_ and 100 mM KCl). The sample was recovered and concentrated back to the starting volume (that is, 250 μl) using an Amicon 30 kDa Molecular Weight Cut Off (MWCO) centrifugal filter. Relaxed state devices were achieved by buffer exchange of the solution with 10 mM Tris pH 8.0 and 10 mM MgCl_2_. For the restriction enzyme nanosensor, the pre-stressed device was separated by agarose gel electrophoresis and purified via the electroelution method^34^. Buffer exchange was achieved by dialysis against optimal BamHI buffer: 50 mM Tris-HCl pH 7.9, 10 mM MgCl_2_, 100 mM NaCl and 0.1 mg ml^−1^ BSA. After dialysis, the sample was recovered from the dialysis unit and concentrated to 20 nM using an Amicon 30 kDa MWCO centrifugal filter. Five units of BamHI enzyme from New England Biolabs were added to 100 μl of the 20 nM closed-conformation device. The mixture was reacted at 37 °C for 60 min on a Peltier thermocycler. For miR-210-sensitive activation, the pre-stressed device was gel purified via electroelution method and buffer exchanged into PBS, to mitigate opening of the device. After dialysis, the sample was recovered from the dialysis unit and concentrated to 100 nM using an Amicon 30 kDa MWCO centrifugal filter. Selective detection of miR-210 was carried out by incubating the origami device concentrated to 100 nM and single-stranded DNA with an identical sequence to miR-210 at three different molar ratios: 1, 5 and 15 equivalents of microRNA to each locking strand. Each mixture was left to react for 2 h at 37 °C on a Peltier thermocycler. As a negative control, the concentrated device was incubated with a random 22-mer sequence (5′-TATGTAGATGATTGCTAGATC-3′) in large excess (50 equivalents to each locking strand) and left to react with the device for 2 h at 37 °C on a Peltier thermocycler.

### Fluorescence measurements

The working solution of the fluorescence assay of the nanosensors experiments was Tris-HCl buffer (50 mM, pH 7.4) or MES buffer (10 mM, pH 5.2), which contained 50 nM of DNA devices and variable concentrations of targets. After an incubation time specific to each design, the nanosensor activity was monitored by recording the emission spectra in the wavelength range of 500 and 600 nm using a spectrophotometer with excitation at 495 nm.

### EGFP fragments

The coding sequences of the large (158 N-terminal amino acids plus a C-terminal cysteine) and small (C-terminal 81 amino acids plus an N-terminal cysteine) eGFP fragments were amplified via classic PCR from a plasmid containing the *eGFP-1* gene from Clontech. Forward and reverse primers used for PCR amplification are as follows: for the large eGFP fragment with C-terminal cysteine 5′-AGTTTCTAGAATGGTGAGCAAGGGCG-3′ and 5′-ATCGCTCGAGTTAGCACTGCTTGTCGGCCATG-3′, and for the small eGFP fragment with N-terminal cysteine 5′-ATCGGATATCATGTGCAAGAACGGCATCAAGGTG-3′ and 5′-ATCGCTCGAGTTACTTGTACAGCTCGTCC-3′. PCR products were cloned into the TWIN-1 vector from New England Biolabs, to yield the C-terminal fusions to the SspDNAB intein. Both fragments were expressed in BL21(DE3) pLys competent *E. coli* cells from Stratagene and purified independently. After intein self-cleavage, the eGFP protein fragments were purified using a S-200 size-exclusion chromatography column. Gel-filtration columns were prepared in a PBS-EDTA buffer at pH 7.5. The two purified fragments were concentrated using an Amicon concentrator 3 kDa MWCO device and mixed at a 10:1 volume ratio with 10 mM biotin-HPDP from Pierce in dimethylformamide. The mixture was then incubated for 2 h at room temperature. Unreacted biotin-HPDP was removed from the biotinylated proteins by using G-25 microspin columns from Amersham Pharmacia Biosciences. Purified biotinylated eGFP fragments were mixed with equimolar amounts of streptavidin and left to react for 30 min at 37 °C in PBS–EDTA buffer. Finally, the coupled N-terminal and C-terminal fragments were combined with biotinylated oligonucleotides (5′-TCGGTCACAGTACAACCGTTATCTACATA-3′-Biotin and 5′-Biotin-TATGTAGATTTTCCCATTGCTCTTAATTGTT-3′, respectively) at a 1:1 molar ratio. Assembly of split eGFP origami device was accomplished in a similar manner as the NA in the presence of two equivalents of 5′-TGGCTTAGCGTTTTATCACAAAATTTAACAATTAAGAGCTGGGA-3′ and 5′-CGGTTGTACTGTGACCGATTGCATGATAGGTGGCTACCAGC-3′ capturing strands. The device was separated by agarose gel electrophoresis, purified via the electroelution method and buffer exchanged into PBS supplemented with 10 mM of MgCl_2_ using a Slide-A-Lyser MINI Dialysis Unit (2, 000 MWCO) from Thermo Scientific. The purified DNA device was then subsequently hybridized with DNA-functionalized split eGFP in a molar ratio of 1:5:5 for 1 h at room temperature. The resulting protein/DNA origami conjugates were purified from uncoupled DNA-functionalized split eGFP by rate-zonal centrifugation. The mixture was loaded to the top of a linear quasi-continuous 15–45% glycerol gradient and spun at 200,000 *g* for 2 h. Fractions were then collected from the glycerol gradient. The 45% fractions containing the split eGFP fragments coupled to origami products were then combined and buffer exchanged with 10 mM PBS buffer pH 7.4, 160 mM NaCl and 10 mM MgCl_2_ using Slide- A-Lyzer G2 Dialysis Cassettes, 20 K MWCO, 3 ml from Thermo Scientific. Split eGFP DNA origami conjugates was concentrated to ∼250 nm.

### Transmission electron microscopy

For imaging, particles were adsorbed onto glow discharged collodion and carbon-coated TEM grids (Ted Pella) and then stained using a 2% aqueous uranyl formate solution containing 25 mM NaOH and visualized at × 68,000 magnification with an FEI Tecnai T12 BioTWIN or a JEOL JEM-1400 TEM, operated at 80 kV in the bright-field mode. ImageJ was used to manually measure the angles of each structure from TEM images.

## Additional information

**How to cite this article:** Ke, Y. *et al*. Regulation at a distance of biomolecular interactions using a DNA origami nanoactuator. *Nat. Commun.* 7:10935 doi: 10.1038/ncomms10935 (2016).

## Supplementary Material

Supplementary Information Supplementary Figures 1-21 and Supplementary Table 1
