# Supplementary material for: Regulation at a distance of biomolecular interactions using a DNA origami nanoactuator
Source: Nat Commun. 2016 Mar 18;7:10935. doi: 10.1038/ncomms10935 (PMC4802031; doi:10.1038/ncomms10935)
Supplement: Supplementary Information — Supplementary Figures 1-21 and Supplementary Table 1 [file ncomms10935-s1.pdf]

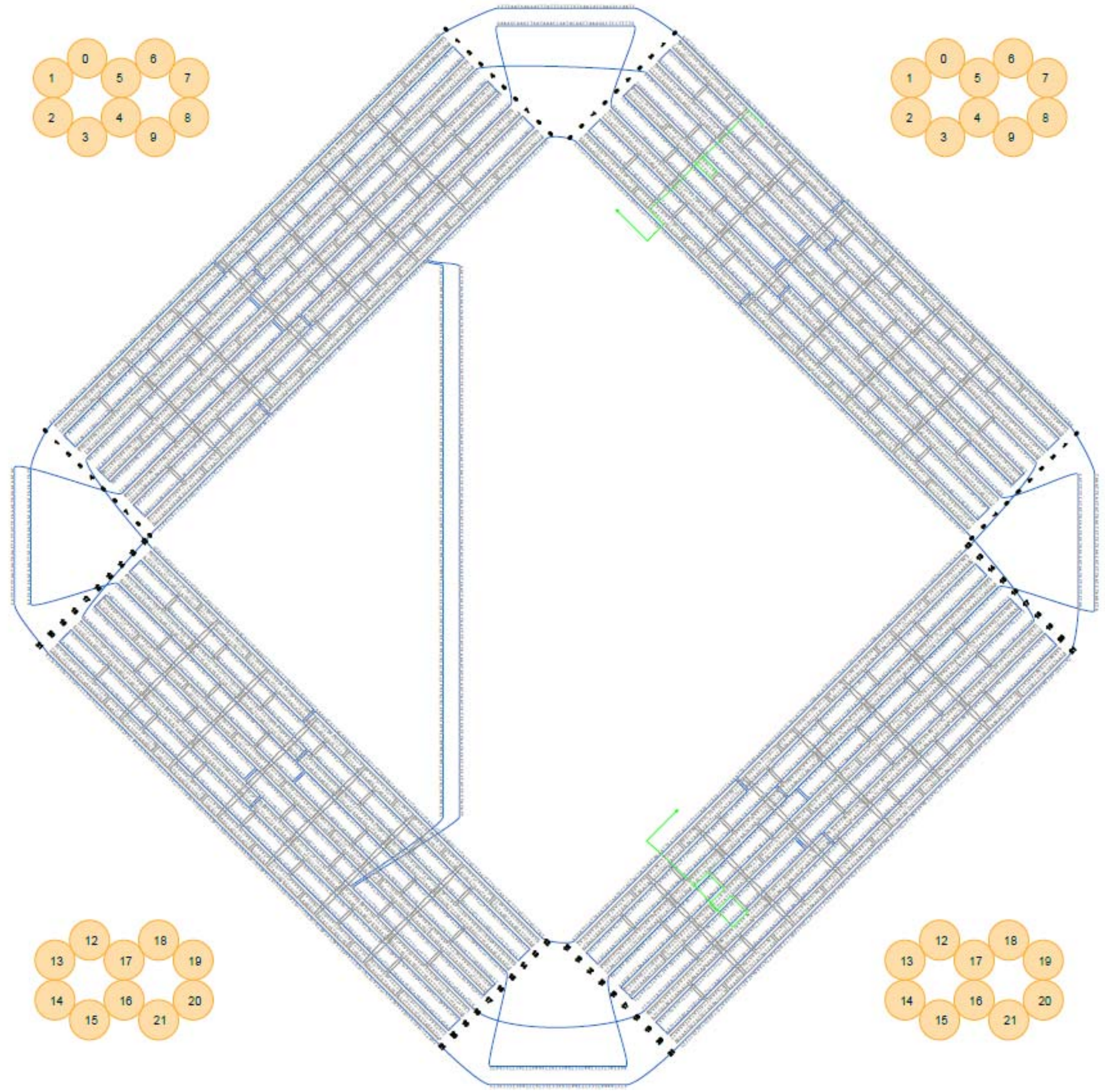

Supplementary Figure 1. Design diagrams of the nanodevice without locking strands. The nanodevice contains four arms. Each arm consists of ten DNA double helices. Insets at four corners show the arrangement of the ten double helices. The numbers in the insets correspond to the numbers in the detailed strand diagram in the middle. The two single-stranded DNAs for capturing cargo molecules are highlighted with green color. The single-stranded scaffold segments that connecting the two left arms and four corners are shown. No 'strut-locking' strands or 'corner locking' strands are included in the diagrams.

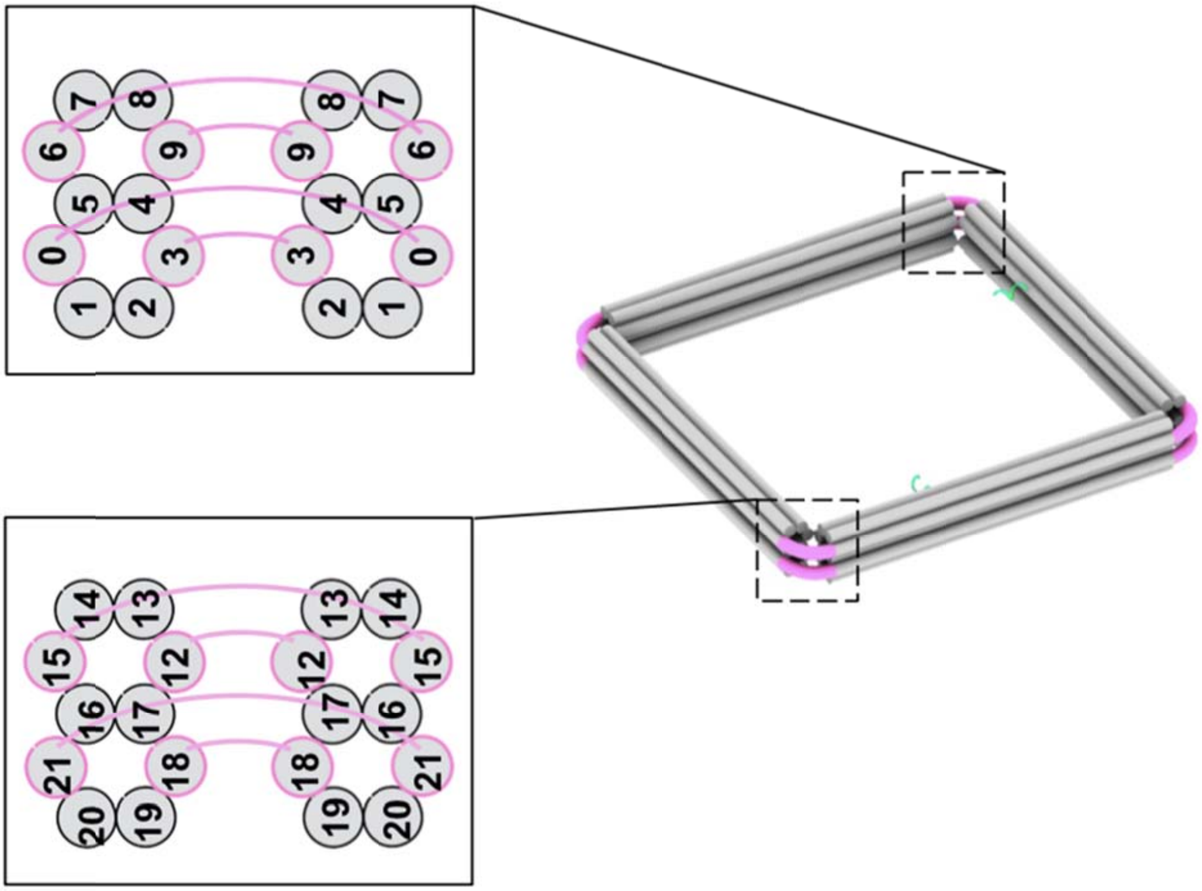

Supplementary Figure 2. The corner connections of the DNA origami nanodevice. The connections at the four corners are formed by the scaffold DNA. The duplexes on the inside (for example, helix9) are connected directly without extra bases in between. Whilst the duplexes on the outside (for example, helix6) are joined together by a 42-base single-stranded scaffold segment. The pink colored parts in the 3D diagram represent the 42-base single-stranded scaffold segments. The two single-stranded DNAs for capturing cargo molecules are highlighted with green color.

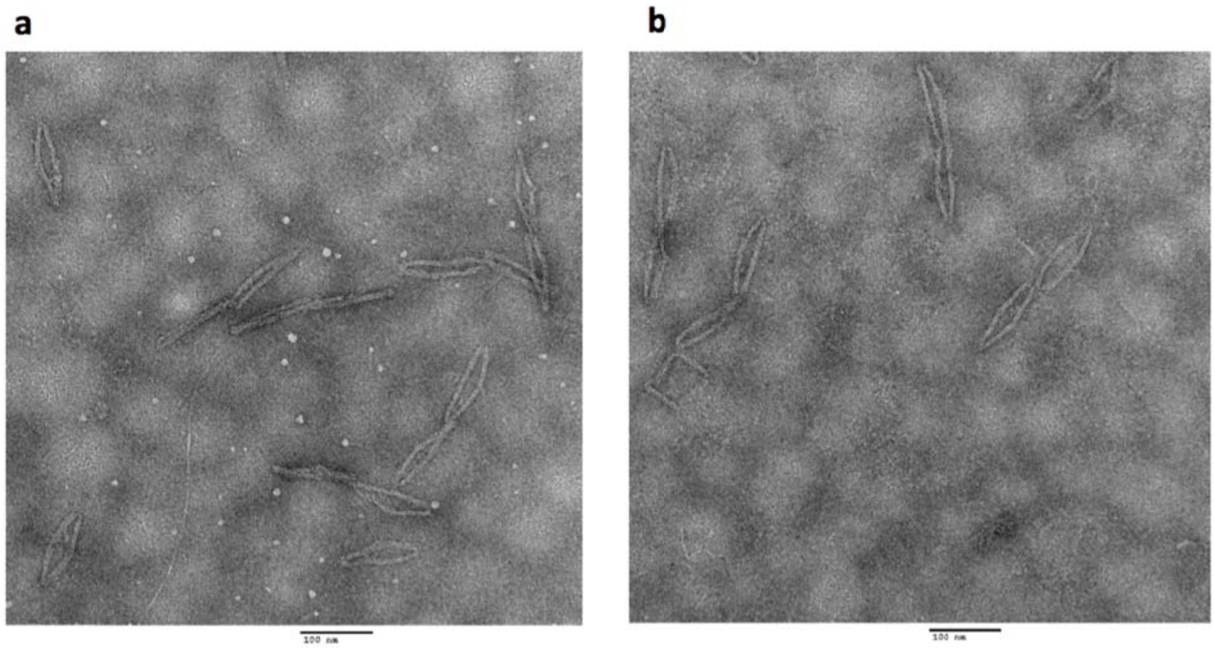

Supplementary Figure 3. Negative-stain TEM Micrographs of NA-UL and NA42 dimer formation. Upper bands from agarose gel in Figure 2a were extracted from gel. Negative-stained TEM micrographs show high prevalence of dimers for a) NA-UL and b) NA42 conformations.

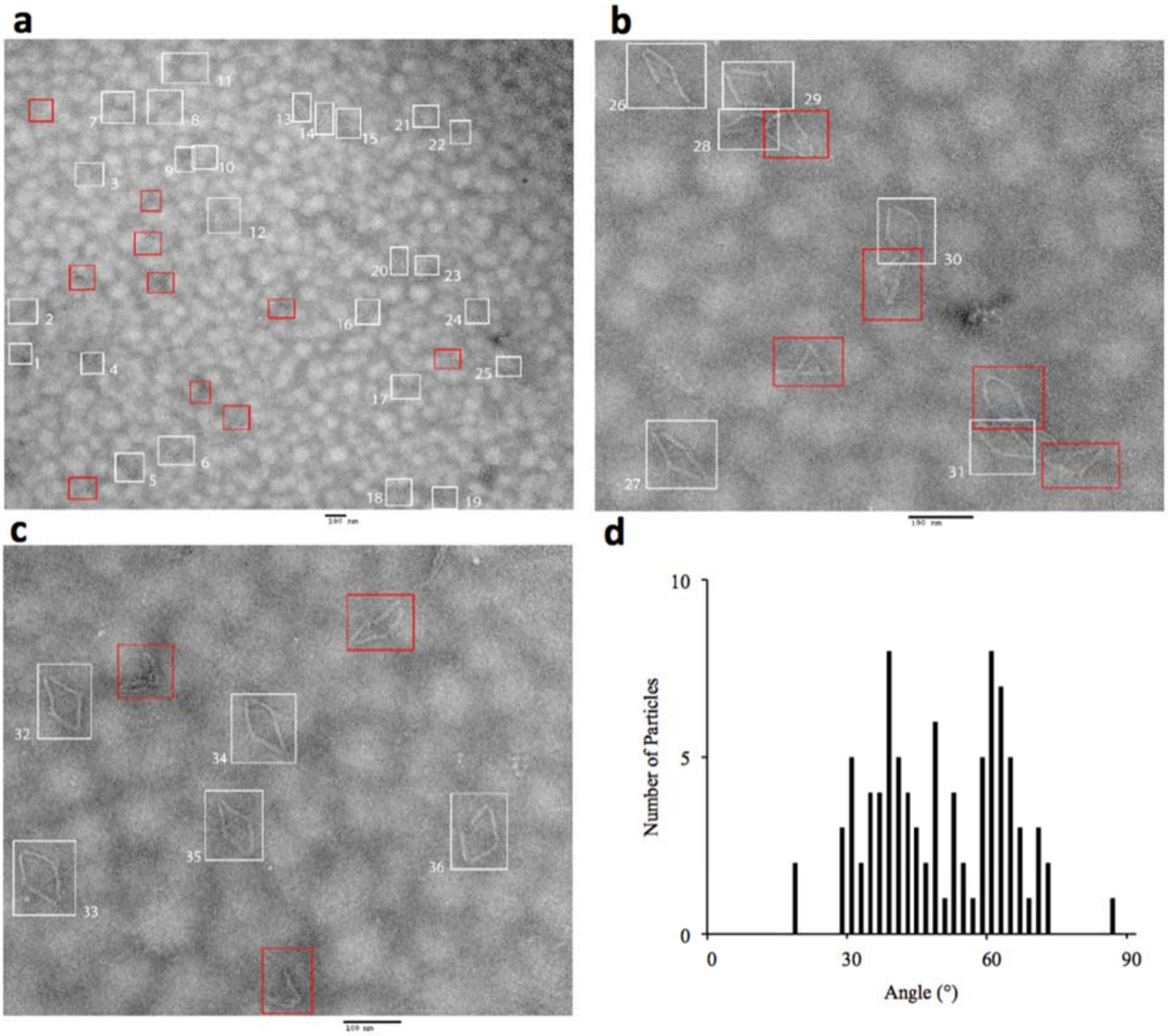

Supplementary Figure 4. Typical field-of-view negative-stain TEM micrographs obtained from agarose gel-purified NA-UL. NA-UL were assembled and objects were purified via agarose gel. a) Zoom-out, typical field-of-view negative-stain TEM micrographs. (b-c) Zoom-in in duplicate of the purified objects. d) Conformational analysis of the TEM experiment results. Histogram of the angular distribution of NA-UL devices. The software ImageJ was used to measure the angles of each structure from TEM images.

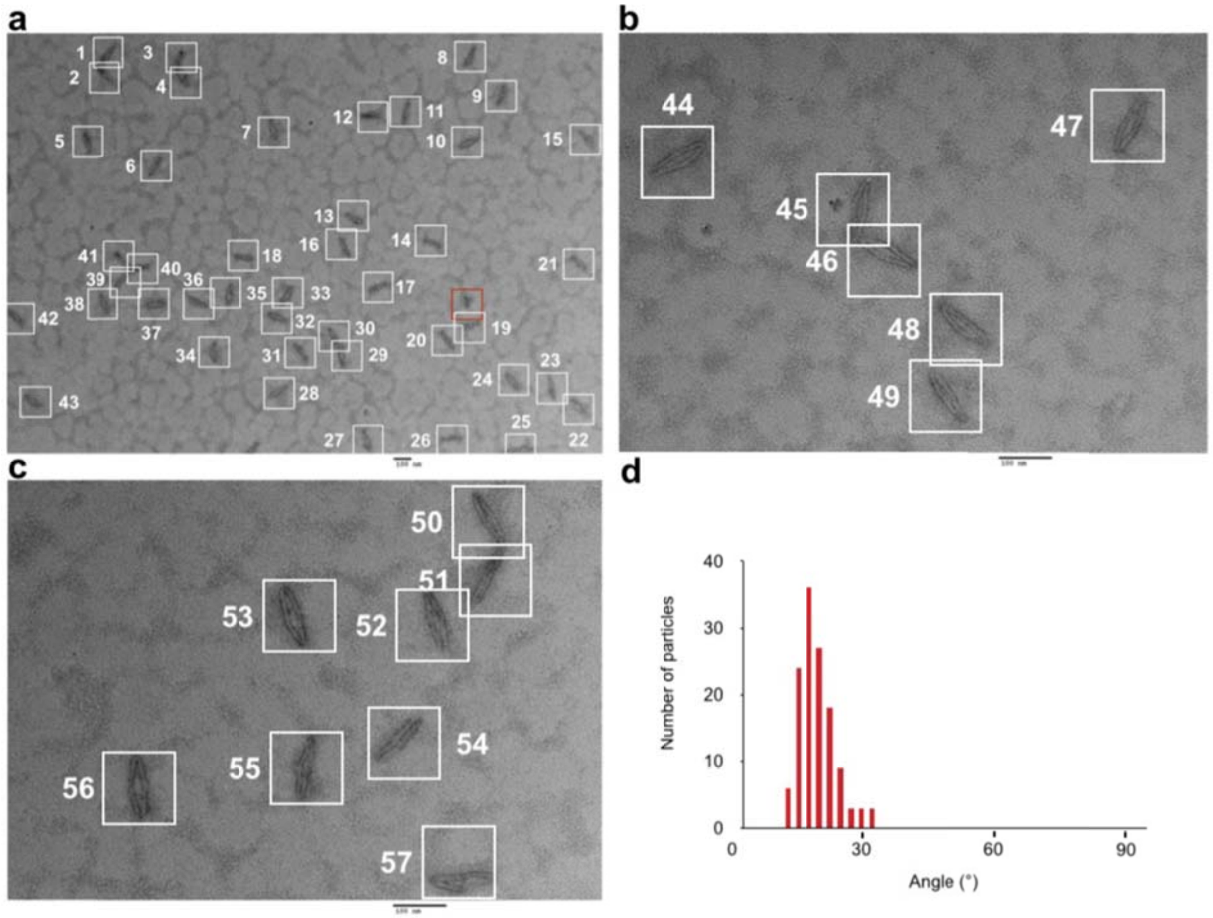

Supplementary Figure 5. Typical field-of-view negative-stain TEM micrographs obtained from agarose gel-purified NA42. NA42 were assembled using strut- locking strands and objects were purified via agarose gel. a) Zoom-out, typical field-of-view negative-stain TEM micrographs. (b-c) Zoom-in in duplicate of the purified objects. d) Conformational analysis of the TEM experiment results. Histogram of the angular distribution of NA42 devices. The software ImageJ was used to measure the angles of each structure from TEM images.

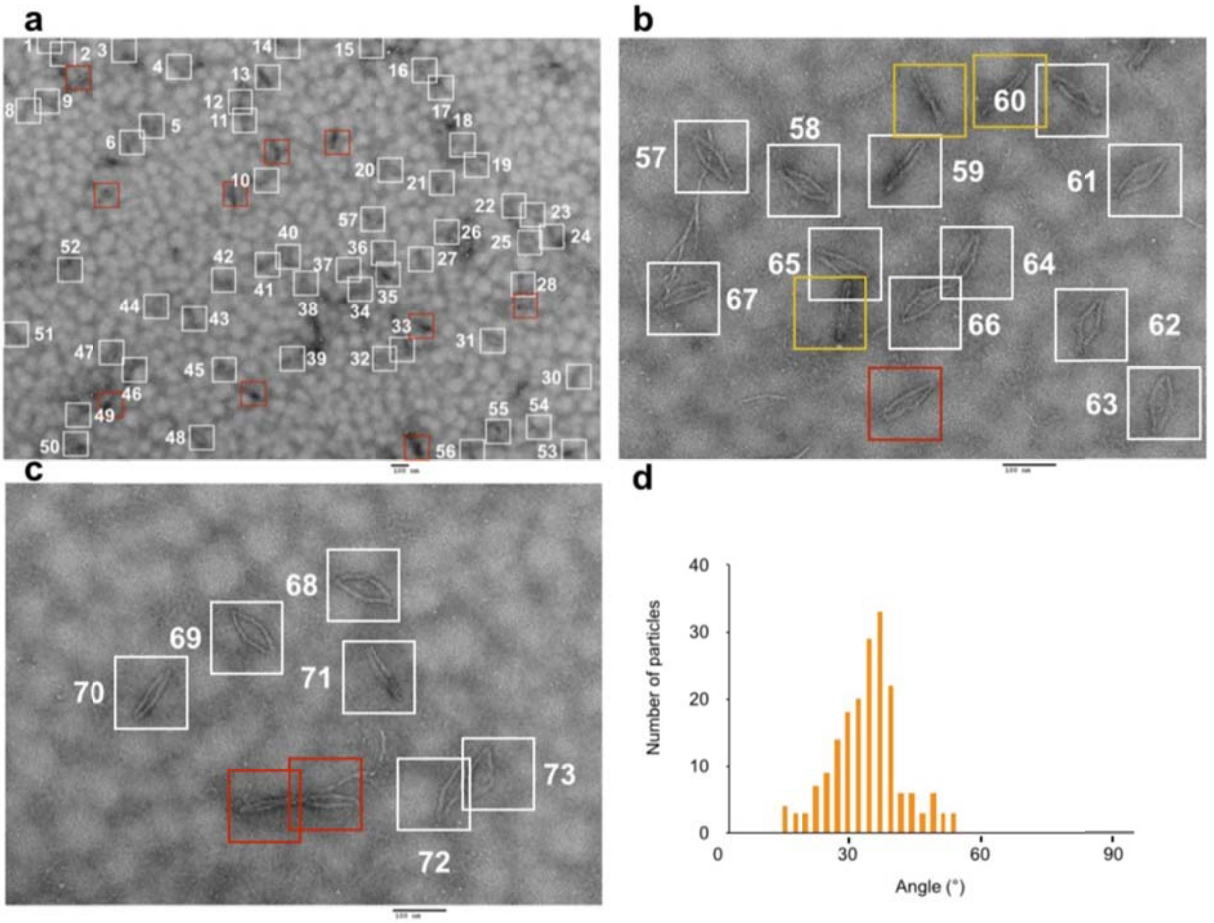

Supplementary Figure 6. Typical field-of-view negative-stain TEM micrographs obtained from agarose gel-purified NA84. NA84 were assembled using strut- locking strands and objects were purified via agarose gel. a) Zoom-out, typical field-of-view negative-stain TEM micrographs. (b-c) Zoom-in in duplicate of the purified objects. d) Conformational analysis of the TEM experiment results. Histogram of the angular distribution of NA84 devices. The software ImageJ was used to measure the angles of each structure from TEM images.

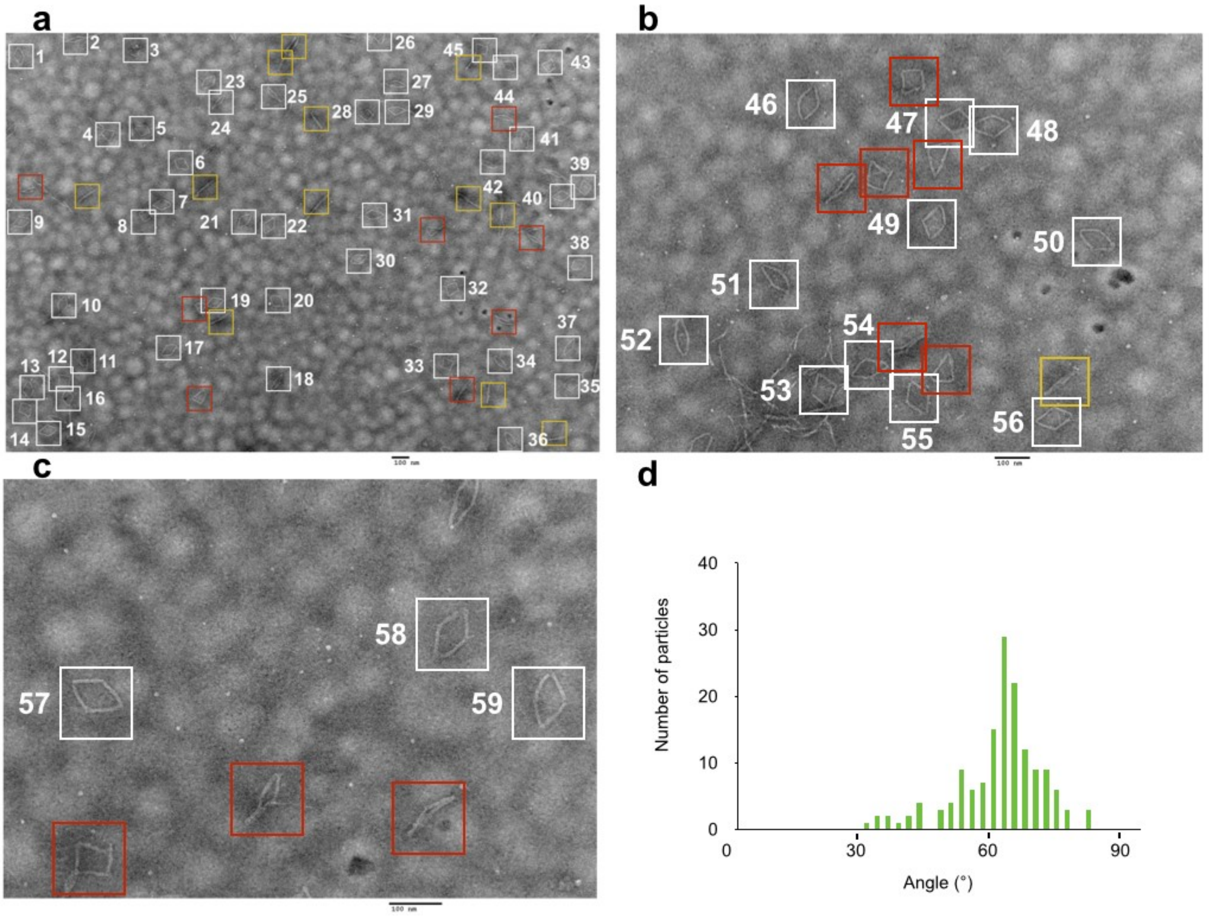

Supplementary Figure 7. Typical field-of-view negative-stain TEM micrographs obtained from agarose gel-purified NA126. NA126 were assembled using strut-locking strands and objects were purified via agarose gel. a) Zoom-out, typical field-of-view negative-stain TEM micrographs. (b-c) Zoom-in in duplicate of the purified objects. d) Conformational analysis of the TEM experiment results. Histogram of the angular distribution of NA126 devices. The software ImageJ was used to measure the angles of each structure from TEM images.

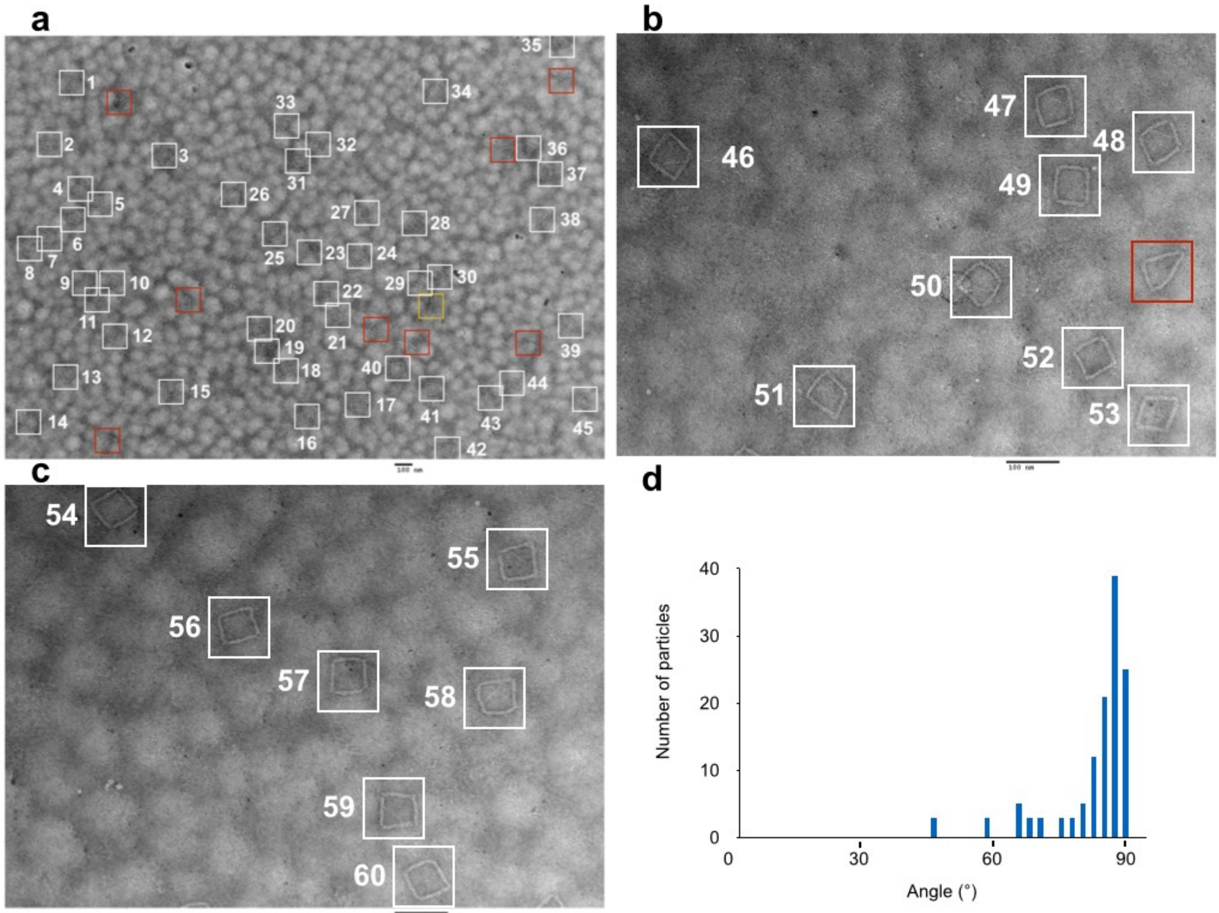

Supplementary Figure 8. Typical field-of-view negative-stain TEM micrographs obtained from agarose gel-purified NA168. NA168 were assembled using strut-locking strands and objects were purified via agarose gel. a) Zoom-out, typical field-of-view negative-stain TEM micrographs. (b-c) Zoom-in in duplicate of the purified objects. d) Conformational analysis of the TEM experiment results. Histogram of the angular distribution of NA168 devices. The software ImageJ was used to measure the angles of each structure from TEM images.

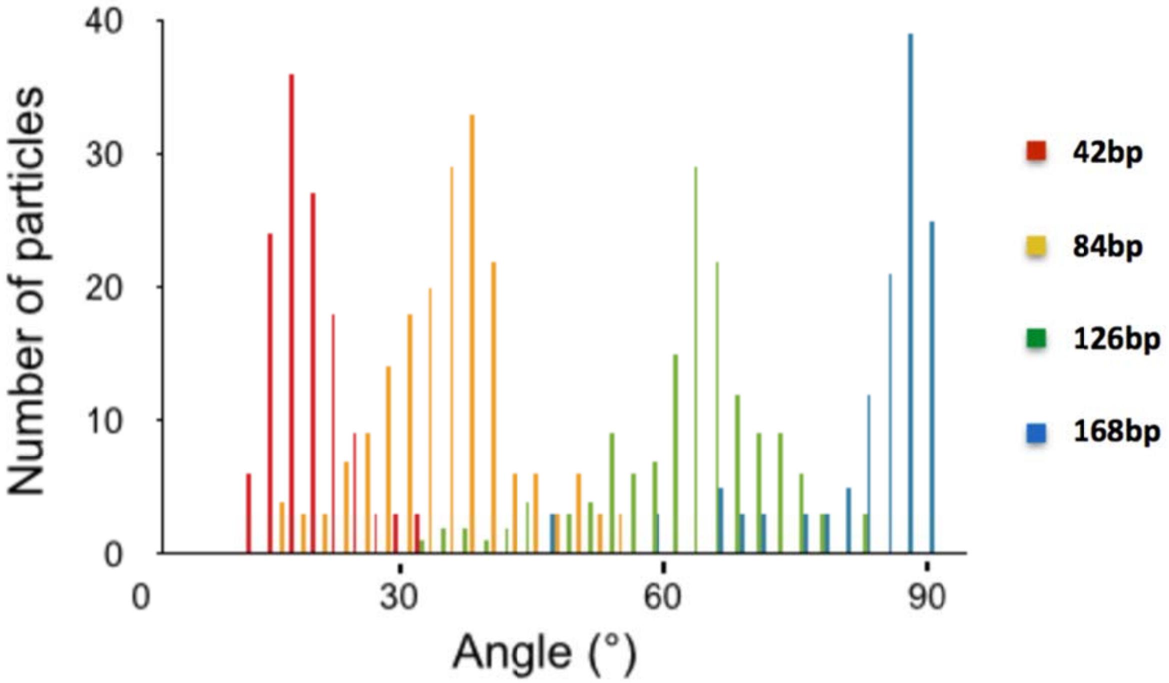

Supplementary Figure 9. Histogram of the angular distribution of the devices. Combined histogram of angular distributions for all nanodevices.

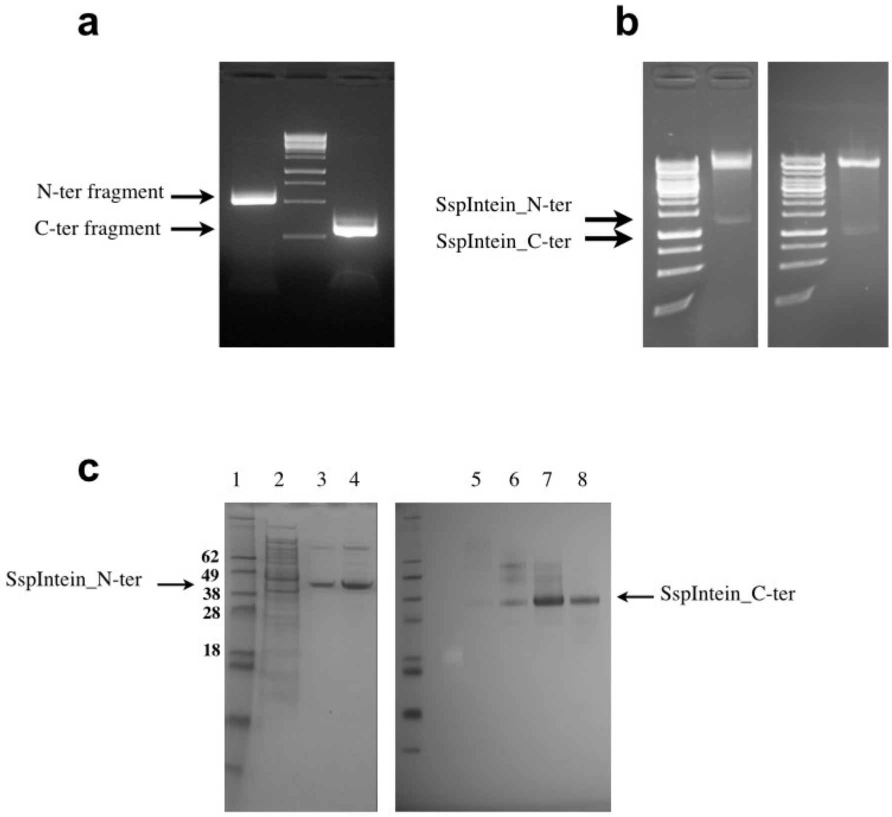

Supplementary Figure 10. Cloning and purification of the EGFP fragments. a, One percent agarose gel of the coding sequences of the large, 158 N-terminal amino acids plus a C-terminal cysteine and small, C-terminal 81 amino acids plus an N-terminal cysteine. EGFP fragments were amplified via classic PCR from a plasmid containing the EGFP-1 gene. b, One percent agarose gel of the hydrolyzed SspDNAB intein sequences before cloning. c, SDS-acrylamide gel of purified Sspintein-Nterminal fragment and Sspintein-Cterminal fragment. Lane 1, molecular weight ladder, lane 2 and 5 E.coli over-expression, lane 4 and 8 purified Sspintein proteins.

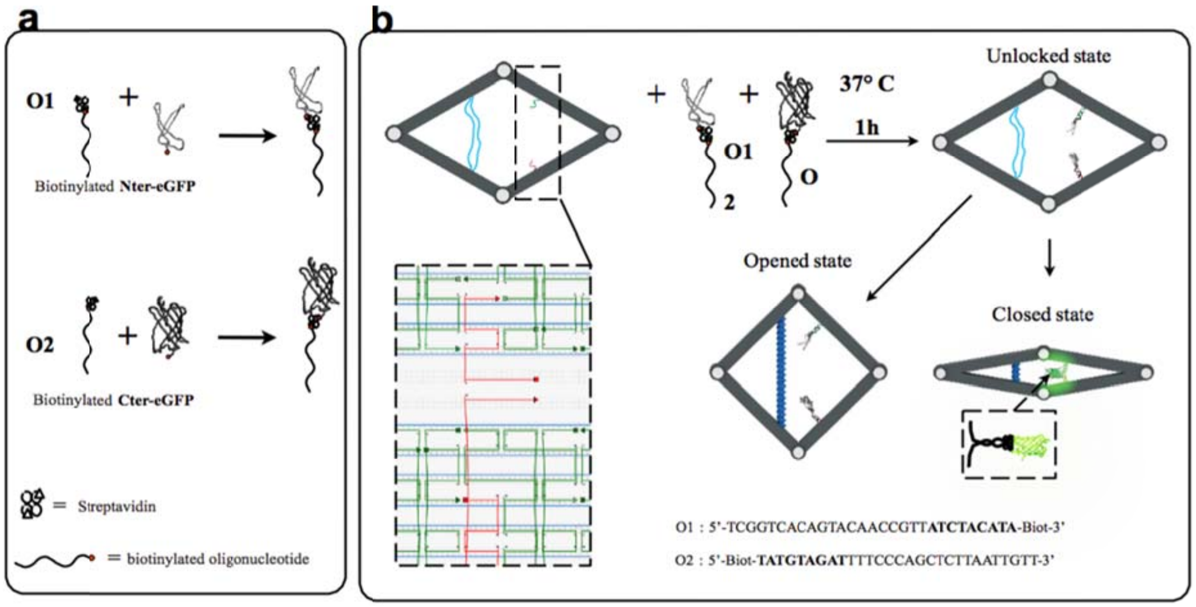

Supplementary Figure 11. Split eGFP constructs and assembly. a The eGFP fluorescent protein is split in two nonfluorescent fragments, a large N-terminal eGFP fragment and a small C-terminal eGFP fragment. Each protein fragment is linked to an oligonucleotide (O1 or O2) via biotin: streptavidin interactions. Oligonucleotides O1 and O2 are linked to N-terminal eGFP fragment and C-terminal eGFP fragment respectively. **b** The two nucleoprotein constructs are associate to the DNA nano-device by sequence-specific duplex DNA formation at 37C for 1h. Purified split eGFP nano-device conjugates is stabilized in open state by adding NC168 strut-locking strands or in close state by adding NC42 strut-locking strands set. The eGFP complementation is drive by transient binding of short DNA duplex (9bp). **Estimation of unbinding rates.** We exploits the transient binding of short DNA duplex to drive the eGFP complementation. This offers the advantages of increasing the complex specificity with an adjustability of on and off times. The off rate for unbinding of two strands is approximately exponentially dependent on the length of the duplex. Dissociation rate of $k_{off} = 2k_+(n\text{-}N)s^{-(n-N)}$. Koff is strongly dependent on the length of the duplex. For values of -DG_{bp} = 3k_BT and $k_+ = 10^7 s^{-1}$ the off rates are on the order of 1.8 s^{-1} for the 9bp duplexes.

n, short DNA duplexes of length n dissociation N, length of dissociated duplex

s denotes the stability constant for one base-pair: s = k_+/k_- = exp(-DG_{bp}/k_BT)

k_+ and k_- are rates for the formation and breaking of a single base pair. 7

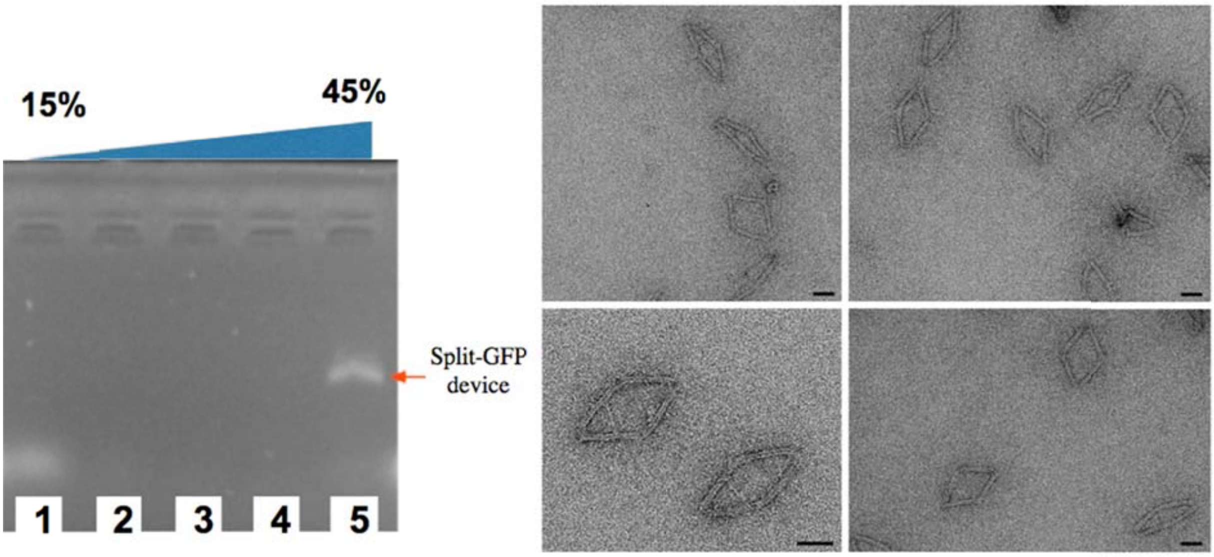

Supplementary Figure 12. Agarose gel electrophoresis analyses of glycerol-gradient purification of the eGFPsplit-device. Left, agarose gel electrophoresis analyses of glycerol-gradient purification of the EGFPsplit-device. Agarose gel electrophoresis analyses of glycerol-gradient fractions collected after centrifugation of the EGFPsplit-device mixture. From the left the fractions collected from top to bottom of the gradient, with fraction 1 the lightest. Right, typical field-of-view negative-stain TEM micrographs obtained from glycerol-gradient purification of the EGFPsplit-device EGFPsplit-device. Scale bar, 50 nm.

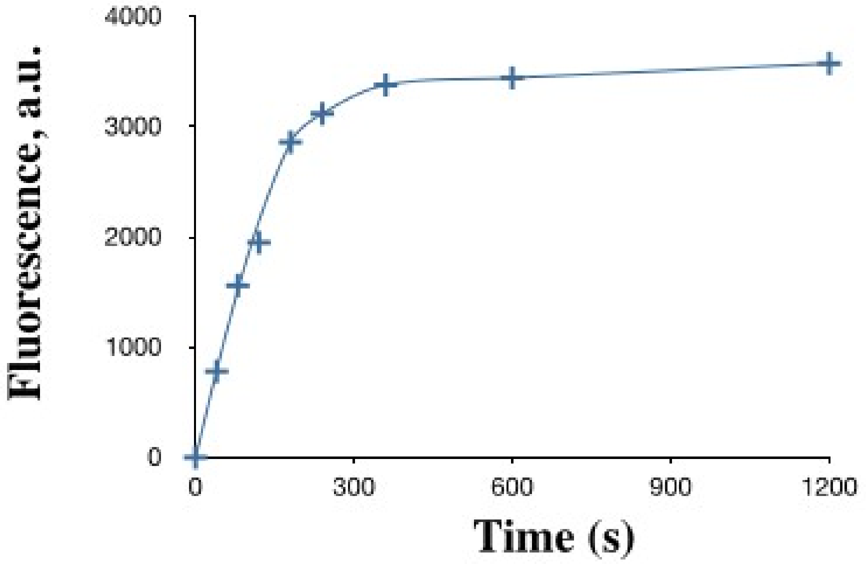

Supplementary Figure 13. Kinetics of reassembled eGFP. Fluorescent responses at 524nm of the split eGFP system upon closed conformation of the device in 10 mM PBS buffer, pH 7.4, 160 mM NaCl and 10mM MgCl2.

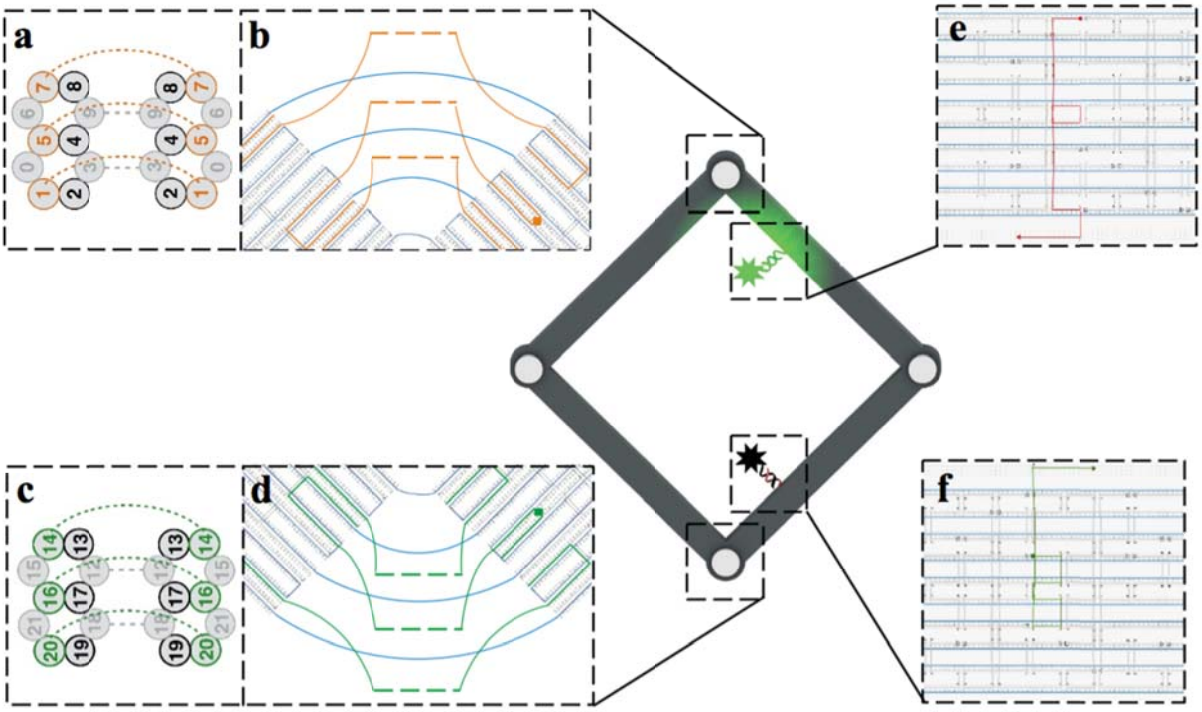

Supplementary Figure 14. The connection patterns that were implemented in the DNA nano-sensor device. The connections at the top corner (orange) and the bottom corner (green) are formed by staple strands and their complementary strands. The two single-stranded DNAs for capturing FAM and BHQ-1 molecules are highlighted with red and green colors respectively.

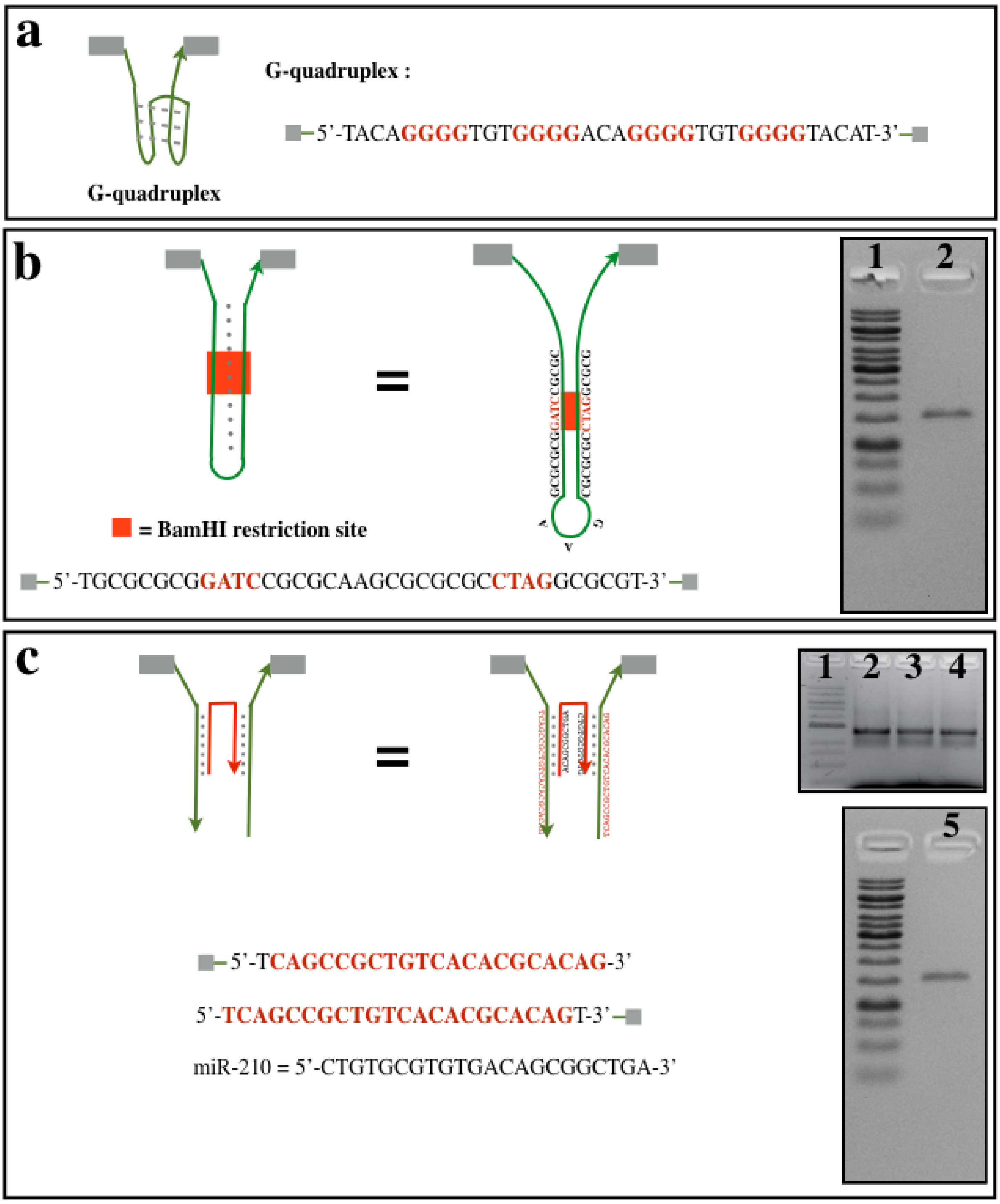

Supplementary Figure 15. Detection mechanisms of the nanosensor device. The lock system was designed to recognized three different systems. **a** A buffer-sensitive activation with a G-quadruplex as sensing elements. **b** An enzyme-sensitive activation containing a specific palindromic sequence of nucleotides which is recognized by the enzyme BamHI. **c** A small molecules-sensitive activation, the micro RNA miR-210 which is a sensor for hypoxic stress during tumorigenesis. Agarose gels: 1, 1kb ladder. 2-4, device before purification. 5, device after purification.

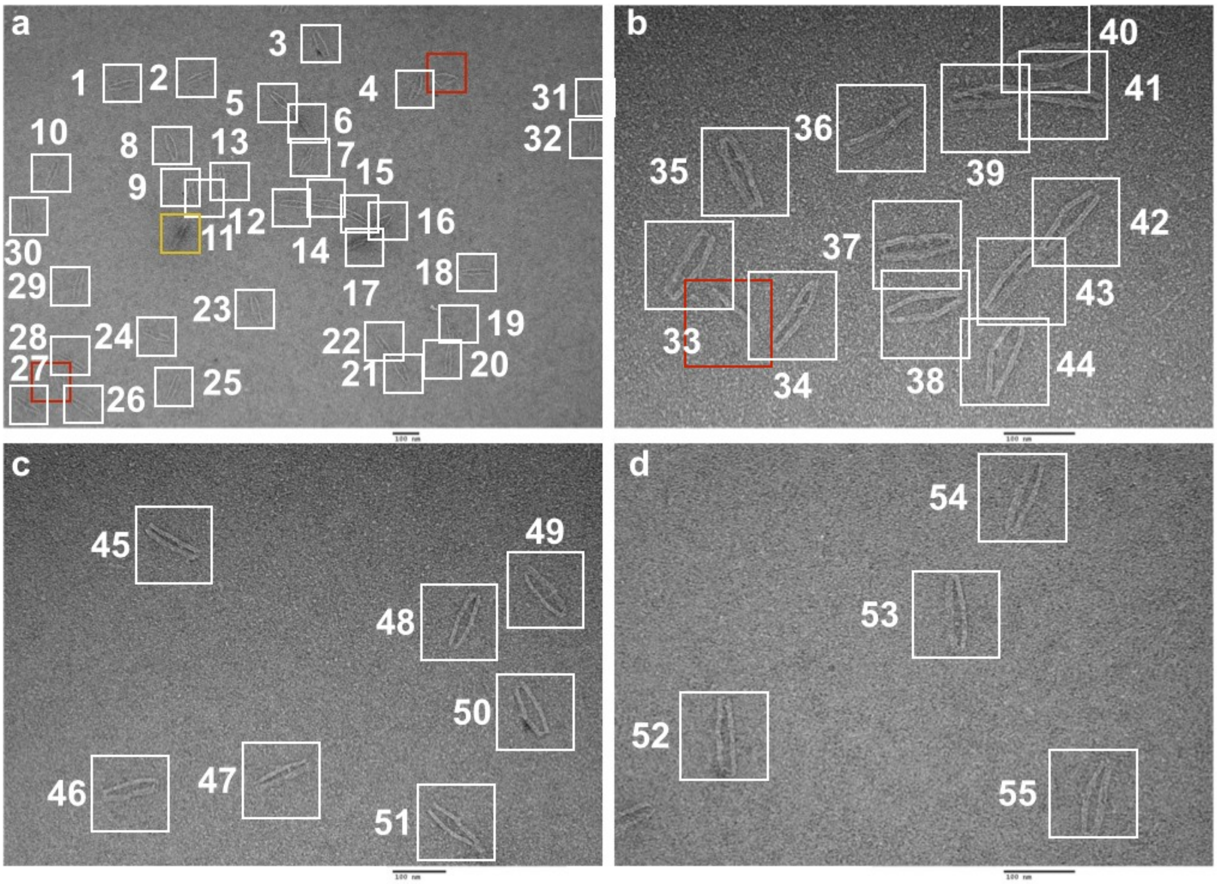

Supplementary Figure 16. Typical field-of-view negative-stain TEM micrographs obtained from purified closed potassium-sensitive device. a) Zoom-out, typical field-of-view negative-stain TEM micrographs. (b-c) Zoom-in in triplicate of the purified objects. The software ImageJ was used to measure the angles of each structure from TEM images.

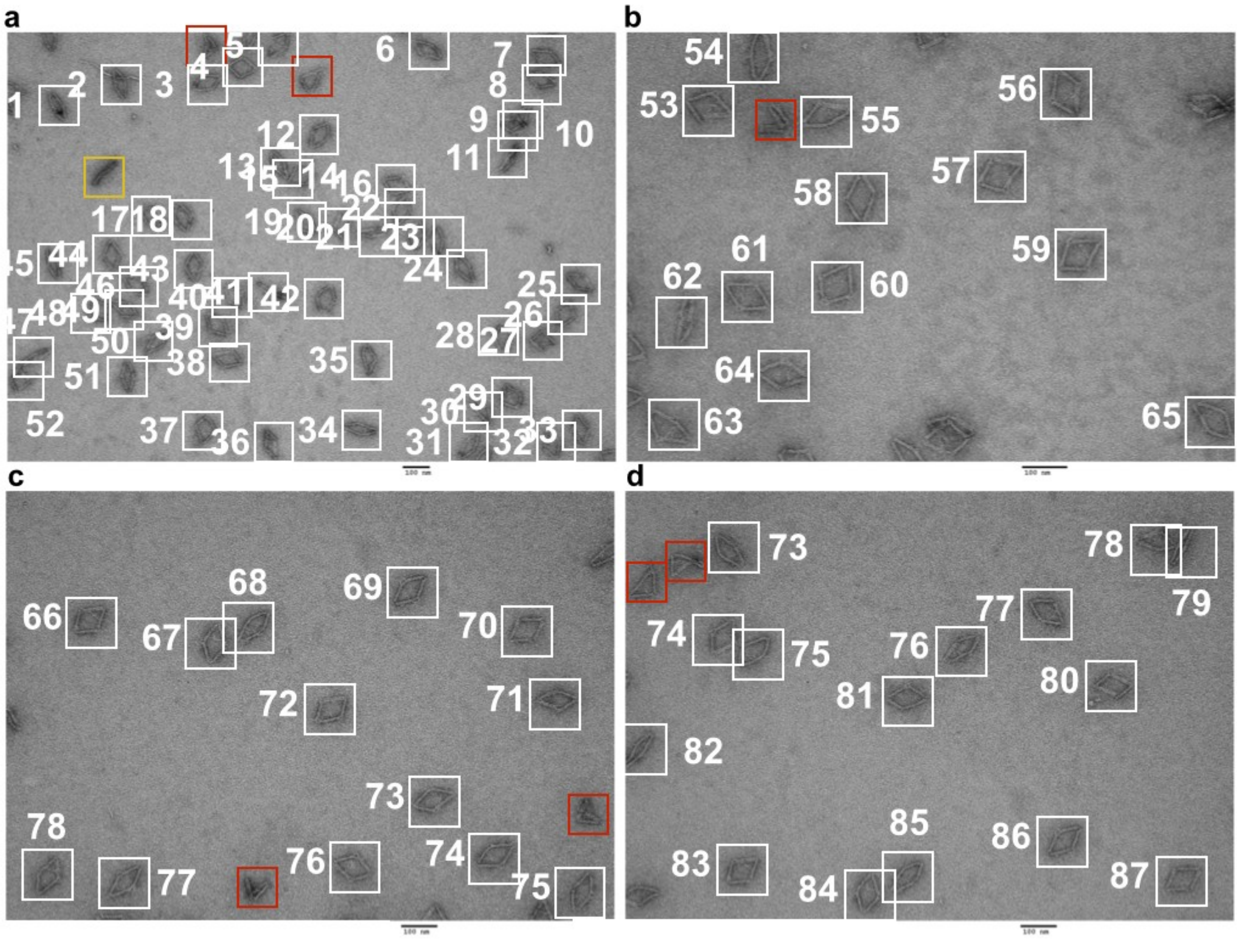

Supplementary Figure 17. Typical field-of-view negative-stain TEM micrographs obtained from purified opened potassium-sensitive device. a) Zoom-out, typical field-of-view negative-stain TEM micrographs. (b-c) Zoom-in in triplicate of the purified objects. The software ImageJ was used to measure the angles of each structure from TEM images.

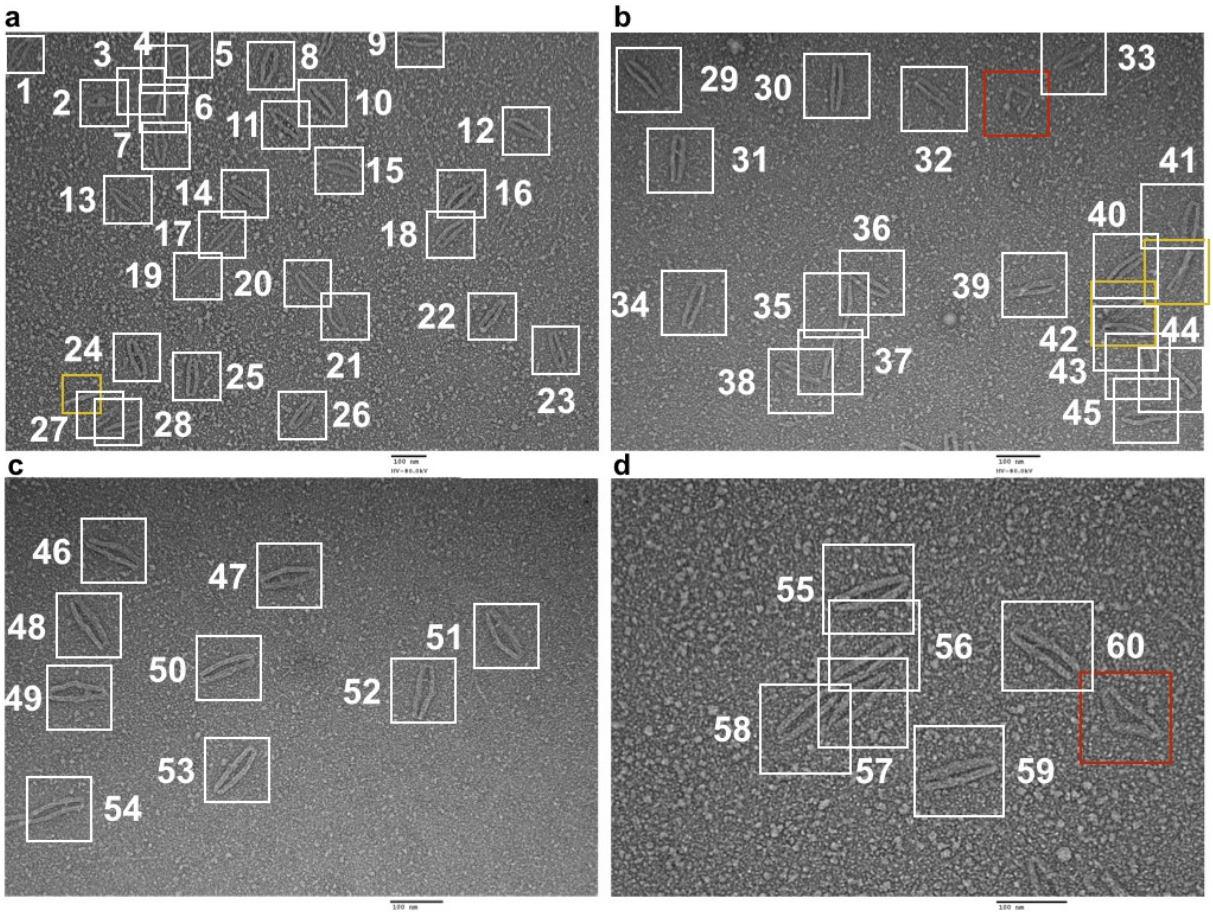

Supplementary Figure 18. Typical field-of-view negative-stain TEM micrographs obtained from purified closed BamHI-sensitive device. a) Zoom-out, typical field-of-view negative-stain TEM micrographs. (b-c) Zoom-in in triplicate of the purified objects. The software ImageJ was used to measure the angles of each structure from TEM images.

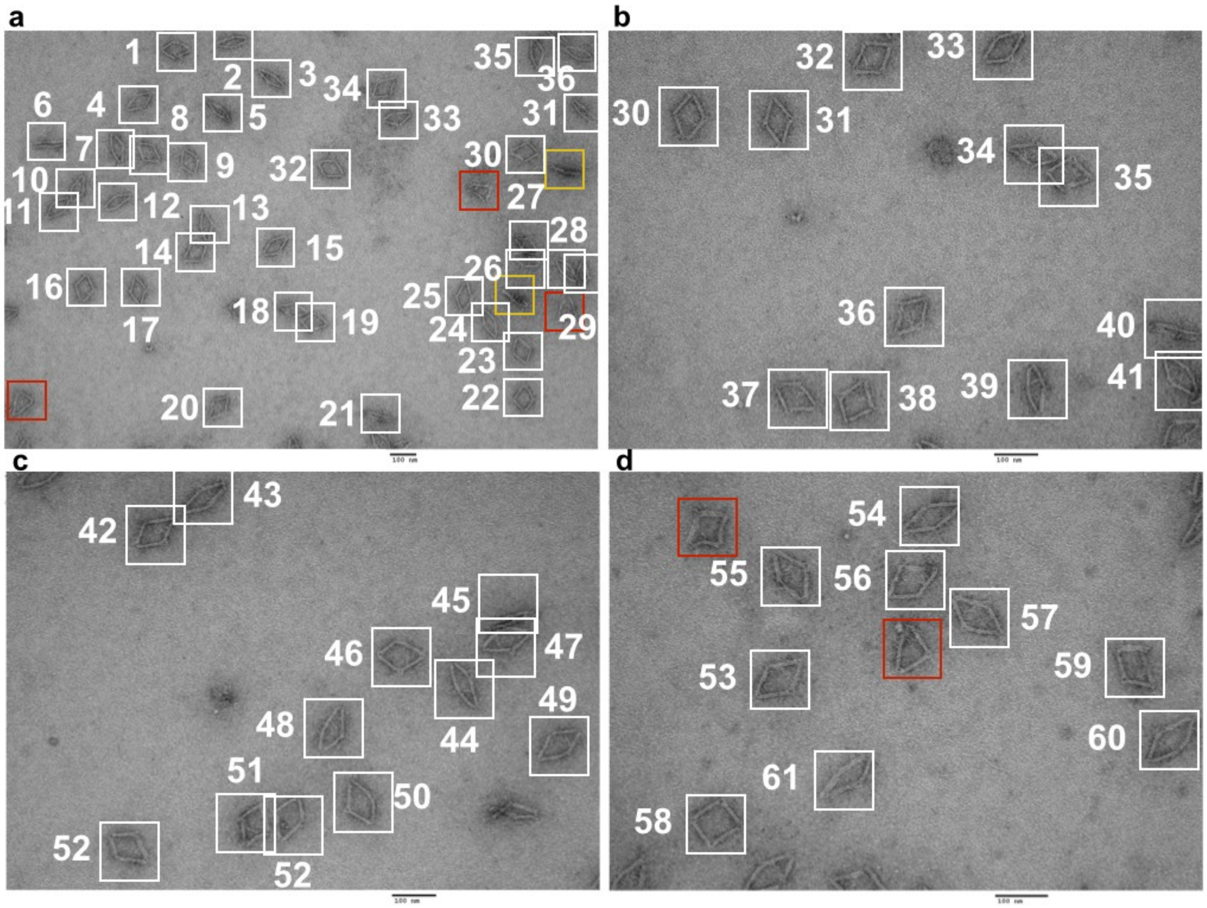

Supplementary Figure 19. Typical field-of-view negative-stain TEM micrographs obtained from purified opened BamHI-sensitive device. a) Zoom-out, typical field-of-view negative-stain TEM micrographs. (b-c) Zoom-in in triplicate of the purified objects. The software ImageJ was used to measure the angles of each structure from TEM images.

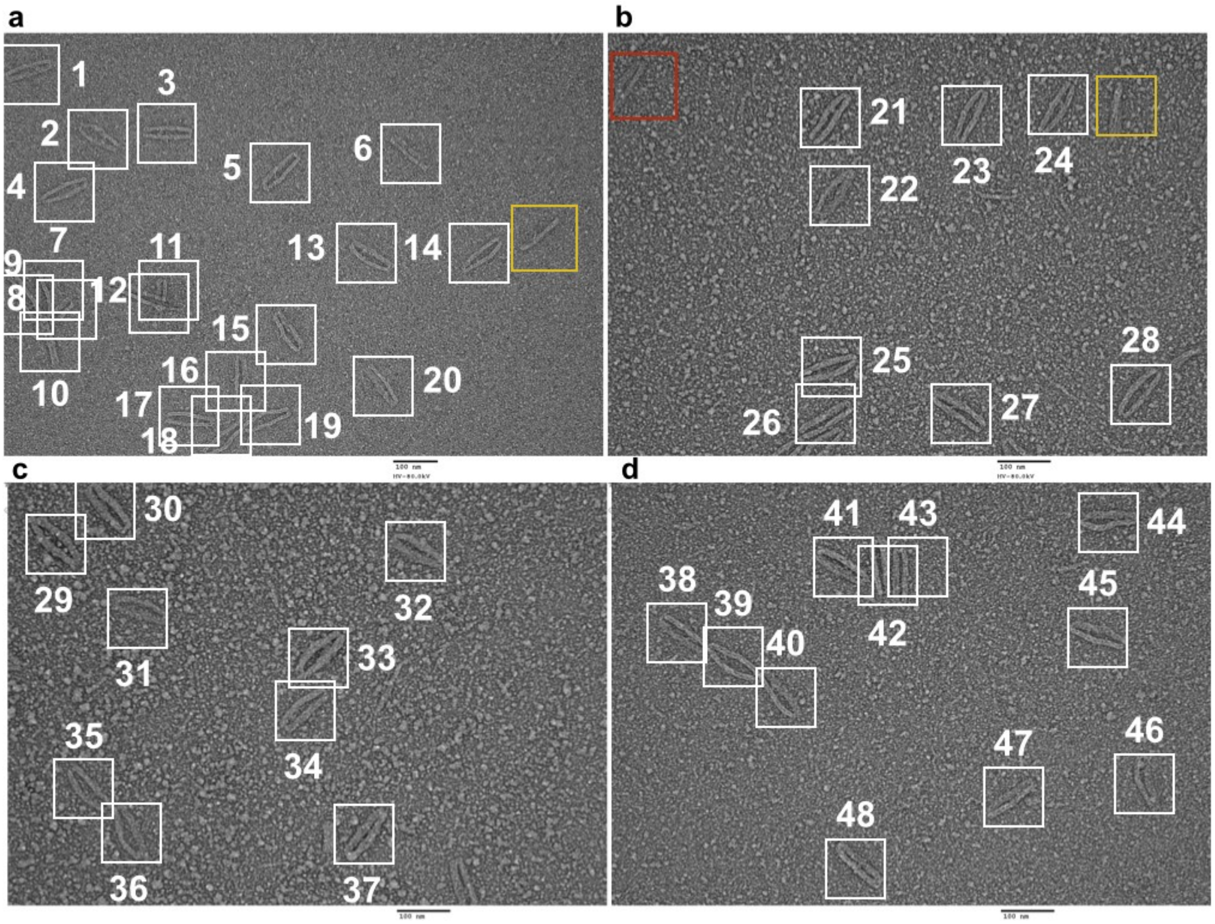

Supplementary Figure 20. Typical field-of-view negative-stain TEM micrographs obtained from purified closed miRNA-sensitive device. a) Zoom-out, typical field-of-view negative-stain TEM micrographs. (b-c) Zoom-in in triplicate of the purified objects. The software ImageJ was used to measure the angles of each structure from TEM images.

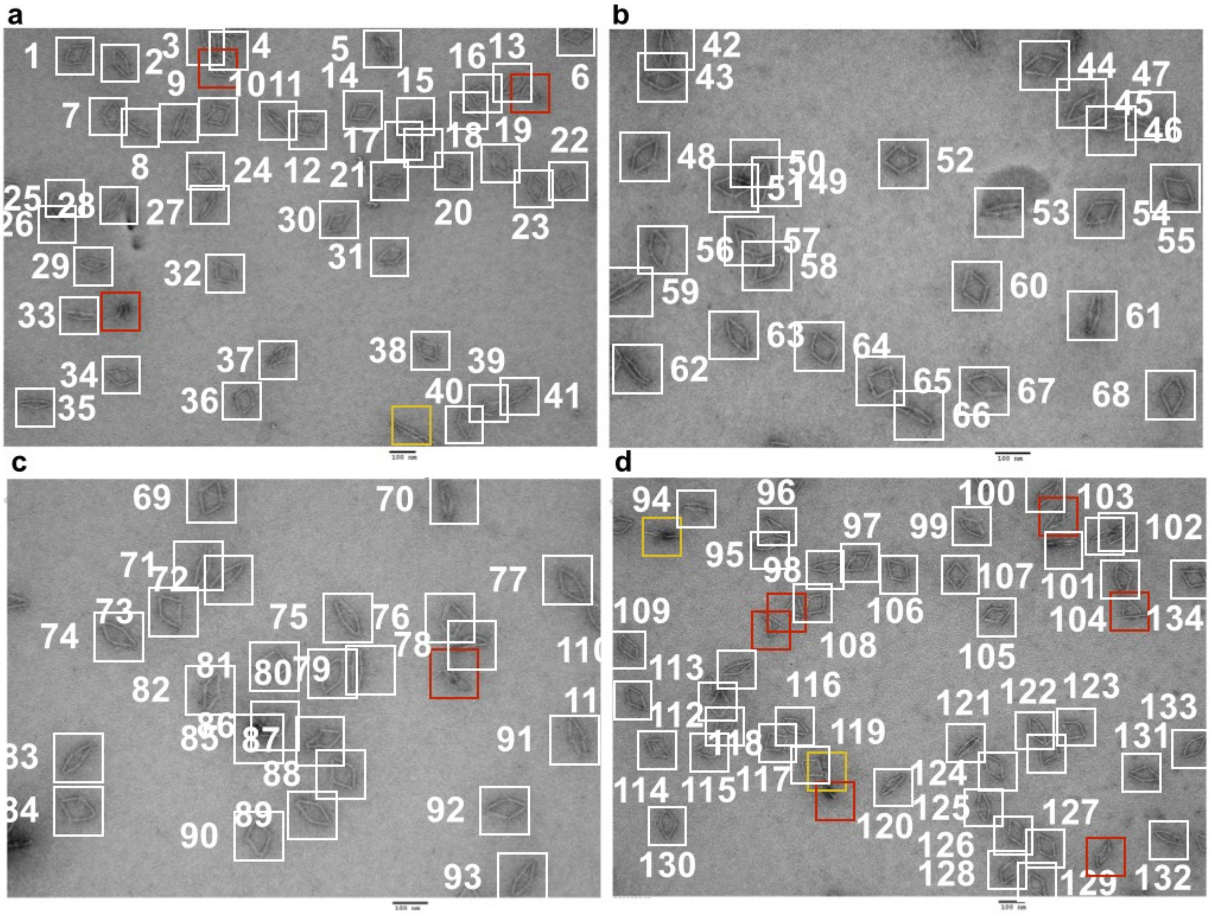

Supplementary Figure 21. Typical field-of-view negative-stain TEM micrographs obtained from purified opened miRNA-sensitive device. a) Zoom-out, typical field-of-view negative-stain TEM micrographs. (b-c) Zoom-in in triplicate of the purified objects. The software ImageJ was used to measure the angles of each structure from TEM images.

Supplementary Table 1. Sequences of the staple strands.

5'end	Sequence	Description
6[97]	TGCTGCCAGCTGTTTGATGGTGGTTCCGTAGATGGTAAAACA	Core
0[139]	ATACATGCAGCCAGCTTCGCTCTCTTTTTAAGAAATTAAACGTCAGCAG	Core
12[55]	CTAATGAATCAGAACAAACAATCTATCATAGGGCGAGTTGAGCTATCAT	Core
9[308]	CAGCAAAGAAGATAGAACCCTTCTGACCTTGGCAGAATTATCATATAACAATTTCA	Core
12[139]	CATAAAGAATGTTTAAATCAACGTACTATGGTTGCTACCAGTACGAGTA	Core
0[55]	TCTATTTGCAAGCGTTTCTTTGCGTCTGGCCTTCCTTTCATCAGGTCAC	Core
21[308]	TTCTTTGTCATCATGAAGTATTAGACTTTTAGGAGGAATACCACAACATAATTTAC	Core
21[208]	AGGAACGGTACGCCAGAATCCTGTTAGAACGAAATAA	Core
21[119]	GCCGCTAGAAAAATGCCCTGACGAGAAAAACCGGAATAGTAACTAAATCTTTTAGA	Core
21[19]	TGGACTCCAACGTCAAAGGGCGACATTCAACGAGGCA	Core
2[375]	TTAAATAAGAATAAACACCGGAATCAATACCGAAGACTACCTTTTTATTGGGAA	Core
0[328]	TTGGGTTACCGTCATAGCAGCTCTAAAGCATCACCATACCGAGTAAGAA	Core
0[375]	TCAAAATCATAGGTCTGAGCCGTGTGATAAATAAGGCG	Core
1[19]	TGTTCTTCTAAGTGGTGAGAAGCCAGGGTGGA	Core
1[49]	CACGACTGCCAGTGTCGTGCCAGCTGCAGGTGGTTGTCCACGCAAAATC	Core
1[133]	CCTGCCTGGCGGATCCACCCTCAGAACCCGGGCCTCTTTCCGCGACAAT	Core
1[208]	CAGAGCCACCACCGGATCAAAATCACCGGAAC	Core
1[238]	AGCCGCCGCGTTTGCATGTACCGTAACACCCTCATAAACAACGAATAAT	Core
1[322]	TCTTCTGTTAGTATTTAACAACGCCAACCCATCGACCGACTTATGGATT	Core
2[41]	TTCTCAGTGTGAATCCCTGGAGTGACTCTATGATACC	Core
2[83]	TTCCCAGTGTCAACCTTATGTCAGTGCCGGAACGCCAGGGTT	Core
2[125]	GTCGAGAAACAGTTATGATACAGGAGTGGGCCTCACGAAAGGAACGGAG	Core
2[186]	ACTCCTCAAGAGAAGGATTAGGATTAAAACATGCTCTGAATTTACCGTTCTGGT	Core
2[230]	TTCATAAACCGCCTCAGGAGGTTGAGGCAGGTCAGAC	Core
2[272]	GGCATTTAGAGCCACCACCAAGAACGCGCACGCGTTTTCATC	Core
0[272]	AGCCGCCACCAGAAAGTTTCACATTCCACCTTATT	Core
0[286]	CGCAAGAAGGTAAATAGCGTCCCTGCAACAGTGCCCGGTCAGACATTCT	Core
3[19]	TCACTGCCCGCTTTCCAGTCGGGTGAGACGCTGAGAGAGAATAGCCCGAGATAGGGTTGA	Core
3[77]	AACGCGCGGGGAGTTGGGTAATAGGTGTGCTGCAAACGACGACGGTCGC	Core
3[119]	GAGGTTTCAGCTGGGGAAGATCCCACGCATAACCGAAAGGCCACGAAGG	Core
3[161]	CGCCACCTGTTGGGGCCGGAAGCTTGATACCGATACAGCATCTGAGGAA	Core
3[208]	TTTTCAGGGATAGCAAGCCCAATTCTAAAGTTTTCTGGAAAATCTCCAAAAAAAAGGCTC	Core
3[266]	CAGTACAAACTAACTGTAGACGCTCAACTTGCCTTTATTGACTAATAAA	Core
3[308]	GAGAATCTCAGTAGAAAGGTGATTCACCAGTCACAAGAGATAAAACAGA	Core
3[350]	GAGGCATCCGGAAATTAGAGCTTTGACGCTCAATCCACAGACCATCGCC	Core
4[160]	AAGGGCGAGATAGCCGAACAATTTTTCAGGAACGAGGGTAGCAACGGCTACAGA	Core
4[186]	GCCATTCAGGCTGCGCAACCTCAGAGCCACCACCCTCA	Core
4[349]	CGTCACCACCTCAAATATCAACCTAAAAAATATTTTTGAATGGCTATTAGTCTT	Core
4[375]	ACCATTACCATTAGCAAGGTTTCGAGCCAGTAATAAGA	Core
5[19]	CCTTCACCGCCTGGCCGGCAACAGCTGATTGC	Core
5[84]	AAATCTTGGGACAATGTCCCGCCAAAATGCCCCAGGTATTGGTCTTTGA	Core
5[208]	GACGTTAGTAAATGAATTTTGTCGTCTTTCCA	Core
2[314]	GTTATACTTTAGTTTATATGTAAATGCTATTCATTCGACAGAGAGCCAG	Core
6[55]	CCTTATAATGGGATAACATTAAATGTGAATAATTCTCACCAGAAACCTGCCAAGCT	Core
6[139]	GACAACAGTCACCCGGTAAAATACGTAATCATCGCATTACGCAGTACCGAAGTGCC	Core
6[185]	AGGTGAATTTCTTAAACAACCAGGCAAAGCGCCATTC	Core
6[244]	AATTTTTTATGGTTAACATATAAAAGAAACCCAAAGTAACGAAGGAACCCCATCTT	Core
6[286]	AGAGGGAGGTAGAAAGGAACAACTAAAGACAAAAGAAATACA	Core
6[328]	ATTTACATGAAAGCACGAACCACCAGCATGAAAAAACCGTAAGCCATATCATATGC	Core
6[375]	CTCATGGAAATACCTACATCAGCAAAATCACCAGTAGC	Core
7[19]	TCCGTGGGAACAAACGACAACCCGTCGGATTC	Core
7[42]	GACCGTAAATCAAAAGTTGCAACGCTCGTCATGCG	Core
7[63]	GTTGGTGAAATCGGCTGGTTTAACCCCGCCTTAGTGCTGAATTCACGACATCGGCC	Core
7[105]	GGGAGTTATATATTCAGTATCTACTGGTACAGTGCCCGTATAGGGTTGATACTCAG	Core
7[147]	CGAAAGAGTTGCGCGCACCGCTTCCAGTAACCTATTATTCTGGCGGGGTTCAGAAC	Core
7[208]	TTTATTTTGTCACAATGACACCACGGAATAAG	Core
7[231]	AAATTCATCACGTTTATGGGAGCATTGACCCTCAG	Core
7[252]	GCCAAAGGAATTGCTTTCAACCCACCACGAACCGCCACCCTCTCGGTCATCGTCAC	Core
7[273]	TTCAACCGATTGGGTATTAACACAGCAAACGTAGAGGCGACA	Core
7[294]	GGCCAACCGACCAGGGAAATTGATGCAATTTTTCAAATATATAAATTCTCTTAATT	Core
7[336]	TACGTGGGTCTGAAGAGCCATACCTCCGTTTAATGGTTTGAATAATTACTTAGGCA	Core
8[69]	CTCCAGCTGTAGCAGCGCCAGTTAATGAGTTGTAAAACGACGTAAGTGTCTTCTAA	Core
8[90]	GCAAAAGCGATTATAAGAGGCGGTTTGCCAGGCGA	Core
8[111]	CACCAACAAAGTACGGGATGTATCACCGTATAAGTATAGCCCTTGAGTAAATAAGT	Core
8[153]	GTTTCCAAGTAAGCATCGGTGGCCACCCTTTGCTCAGTACCAATTTCGGAAGCGTC	Core
8[186]	GGCTTTGAGGACTAAAGACAGTTACCAGAAGGAAACCG	Core
8[258]	TACATAATAAGACTCAGACAGCTGAGTTTAGCCCCCTTATTAACCCTCACAGAGCC	Core
8[300]	GGTGAGGACGCTGAATCAAGTAGTAGGGTACCAGTATAAAGCAGAAAACATCCAAT	Core

8[342]	ATTAAAATTGCTGAAATGAAAATGTAATTAGAAAAAGCCTGTACCTAAAGCTTAGG	Core
8[375]	TAATGCGCGAACTGATAGCACCCTCAATCAATATCTGG	Core
9[19]	AACCAATAGGAACGCCATCAAAAGCGAGTAGCGGATT	Core
9[77]	CCCCCAGAATACACGCGCATCGTAACCGTGCATAGGAAAGAG	Core
9[119]	ATTTGTATGCCACTGCTTTTGCGGGATCACCATCGCGCACTCGCTTTTGAATGCCC	Core
9[208]	AGGAAACGCAATAATAACGGAATACGCAAACAATAGA	Core
9[245]	GCATGATAGGTGGCTACCAGC	Core
9[266]	ACGCAGTATGTTCGAGCAACGCCTGTAGGCGGAGTGAGAAGACACTCAG	Core
20[342]	AAAGAAACTGAGTATAATTACATTAATTCAAGCAAGCCGTTTTAGAAACGACGACG	Core
20[375]	AAAAGTTTGAGTAACATTAATCGGCCTTGCTGGTAATA	Core
12[76]	AAAGTGTGGAAGATTCATTGCGTGTAGCGGTCACGTTACAGGAGACGAC	Core
12[97]	AATACTTAGGCTTTAGATCTATAACCACCACACCCCGAACTACTCATTC	Core
0[186]	CCAGAATGGAAAGCGCAGTAAAGTATTAAGAGGCTGAG	Core
12[186]	ACAGGCAAGGCAAAGAATTGTGTAGGTAAAGATTCAAA	Core
12[244]	CTACTAAGCTACAAAGCGAACTTTTTATAATCAGTTACTTCTTTTTCAG	Core
12[265]	GCTGAAATTACCAATCTAAGACCGAGTAAAAGAGTTAATCCTGAATATA	Core
12[286]	ATCAACACGGGAGATATAGAATCACGCAAATTAACGATGATGGATAATA	Core
12[328]	ACAATAAAAGTTACAATTTCAATAACATCACTTGCCCACCAGAATTCGA	Core
12[375]	GAATATAAAGTACCGACAATCCTTATCATTCCAAGAAC	Core
13[19]	ATTCGTAATCATGGTCCGGGTACCGAGCTCGA	Core
13[49]	TGTGAAATCGATAACATTTCACATAAATTCTGGAGAAGCCCCTGTTAAA	Core
13[133]	ACCCTCACAACCGTGAAGCCCGAAAGACTGACCATAGACTGGTACAGAG	Core
13[208]	ACGAGTAGATTTAGTTATTCCCAATTCTGCGA	Core
13[238]	ATTTCGCTTTCATTTTGCGGA	Core
13[322]	GAGCATGTTATTTTTGTAAATCGTCGCTATTTAACAAAATCGTATCTAA	Core
14[41]	GGATCCCATAGCTGAACTCACATTAATTGCGTTGCGC	Core
14[83]	GGGCTTAACACAACATACGGCGGGAGAATTAATGAGTAAACA	Core
14[125]	GATAAATTAAAAATGGTTGTACCAAAAATGCCAGACCCTGACTTAATGC	Core
14[186]	AGGGTGAGAAAGGCCGGAGACAGTCAGAGTAATAGCAAAATTAAGCACCAATAC	Core
14[230]	ACAGTTGTGACCATAGCATTAACATCCAATAAATCAT	Core
14[272]	TATGCAAGCTATATTTTCAAGATAAGTCAAACATGTTTTAAA	Core
14[314]	GGAATCACCATCCTGTTCAGCTAATGCACGCCTGATTTAATGGCAATAC	Core
14[375]	GGGTATTAAACCAAGTACCGCACTCACTGTCTTAAGGTAAAGTAATTGCGAATT	Core
15[19]	ACGAATATAGGGGCCTTGAATCGGATGAACTACCCCGTTTGTTAAATCAGCTCATTTTTT	Core
15[77]	CTGACCTTATCAGGTGTATAAGCAAAAGTTAGCCGGAAGCAT	Core
15[91]	GGTGTTTTGAAGCAAAGCGGAGGAGAGGGTAGCTAGCCTTTAACCCTGT	Core
15[119]	AAAAAGAGTCTTTAGGGGGTATATTCATTACCCAAAAGGCTTCTACGTT	Core
15[161]	TTTAATTAAACAGTTGCGGAAAAAAATGAAAATAGGGGCTTGGAACTGG	Core
15[208]	TTAATTGCTCCTTTTGATAAGAGCTTGCGGCTATTTTTTTATCCCAATCCAAATAAGAAA	Core
0[97]	TTTAACGTGAGGGGGGCGATTACCAAGCGCGAAACCTAAAACGCTTGCA	Core
15[266]	AATATAACCGGTATCGCTAACGAGCGATATTTTGGGGCGCGA	Core
15[280]	GCTCAATCAAAATCAATATATGCCCAATAGCAAGCCTGAACACCTGTTT	Core
15[308]	AATAACCTACCTTTTTGCTTTCACTAACAACTAATGGATTTAATTCCTG	Core
15[350]	CCTTAGACAAGAAAATTCATTTTGAAAGGAATTGATATTAAAGCGGAAC	Core
16[160]	TCAGAAAAGCACGTATAACGTATTTTAAAGATGGTTTAATTTCAACTTTAATCA	Core
16[186]	AATCCCCCTCAAATGCTTTCGAGCTTCAAAGCGAACCA	Core
16[349]	ACAAAATGAAGAACTCAAACTTCATTTTTCCTTTGCCCGAACGTTATTAATTTT	Core
16[375]	AAGATGATGAAACAAACATATCCTTGAAAACATAGCGA	Core
17[19]	GCATGTCAATCATATGGGTAATCGTAAAACTA	Core
17[208]	AAATCAAGATTAGTTGGAGGTTTTGAAGCCTT	Core
18[244]	AAACAGCGCGTAGAGAATAATGGAAGGGAGAAGTGCTCCCGAGTCATTTCCATATA	Core
18[286]	AACTTTTACTCTTTCCAGAGCCTAATTTGTCAGATGATTGTT	Core
18[328]	AATATCTTACAAACAAGGAGCGGAATTAATTAGTATTTGAATTTGCTTCCATCGTA	Core
18[375]	TCAGTTGGCAAATCAACAGTCAATTACCTGAGCAAAAG	Core
19[19]	ACGCCAAAAGGAATTACTAATGCAGATACATA	Core
19[42]	TAGTAAGTTAAATTGTTGATAGTGAGCTTTTCCTG	Core
19[63]	AACCCTCAATATTTAAAAACAAAAGCCTCCGCTCACAATTCCAGCTACGCCGAACT	Core
19[84]	GATAAAAACCAATGACGGAACAACATTACTGCGCGCAAAGGCCCTGGTT	Core
19[105]	AGTGAATATCAACGGAAGTTTCATTATGTTTCAACGCAAGGATAATGCCTTGCATC	Core
19[147]	GTAAATTCAGCCTTATAGCGTATAAAGCTAAATGCAATGCCTAATCACCATCGCGT	Core
19[208]	ATTTGCACGTAAAACACTACCATATCAAAATT	Core
19[231]	AGAAATTCATATTAGCACCCATAGTAGTTAGATAC	Core
19[252]	GTTTAACGCCAGTTCTGAATCAGGTGGCCAATAACCTGTTTACTAAAGTATTGCTG	Core
19[273]	CAGTAACAGTACTAGCAATTCATCAATACTGTCCAGGCTTATTGCTGTA	Core
19[294]	CATTTGAAGATTAGACGGATTGAACGCGAGAAAAATAATATCTTACCGCGTGAGTG	Core
19[336]	CAACTCGGGAAGGTCGCAGAGCTGTCCACAATCAATAATCGGTCGAGAAAATTTTC	Core
20[69]	ATTCATCCTGGCAACTGAGAGCATTTCTTGGTGCTTGTTACCTTGTTATGGGGTGC	Core
20[111]	AATAAAAGCCGCGCTATTATAGTCAGAGTGCAAAATAACAAA	Core
20[153]	CTCATTATTTGACGACGAGAATTCAAATATCAATATGATATTTATATTTCTCAGAG	Core
20[186]	TTGTGAATTACCTTATGCGGCTTTCCTCGTTAGAATCA	Core

20[258]	TGGATTAGAGGCCAACGCGAGGAGCTTAACGGTGTCTGGAAGAAATGGTATCAATT	Core
20[300]	ATTATCACGTTGTAGAAACAGTACATGAAACAATAAGCCGTC	Core

28[188]	GATAAATTGTGTCGAAATCCGTCATCAAGAGTAATCTTGACA	42BPLock
29[21]	AACATAAAAACAGGGAAGCGCAATAGCTATCTTACCGAAGCC	42BPLock

29[21]	AACATAAAAACAGGGAAGCGCTCATCAAGAGTAATCTTGACA	84BPLock
29[42]	ATTAGACGGGAGAATTAACTGAGCAAGAAACAATGAAATAGC	84BPLock
28[188]	GATAAATTGTGTCGAAATCCGAATAGCTATCTTACCGAAGCC	84BPLock
28[167]	CGACCTGCTCCATGTTACTTAGGCGCATAGGCTGGCTGACCT	84BPLock

29[63]	AACACCCTGAACAAAGTCAGATGAGTTAAGCCCAATAATAAG	126BPLock
28[188]	GATAAATTGTGTCGAAATCCGAATAGCTATCTTACCGAAGCC	126BPLock
28[167]	CGACCTGCTCCATGTTACTTAAGCAAGAAACAATGAAATAGC	126BPLock
29[21]	AACATAAAAACAGGGAAGCGCTCATCAAGAGTAATCTTGACA	126BPLock
28[146]	GCCGGAACGAGGCGCAGACGGAGATGAACGGTGTACAGACCA	126BPLock
29[42]	ATTAGACGGGAGAATTAACTGGGCGCATAGGCTGGCTGACCT	126BPLock

29[63]	AACACCCTGAACAAAGTCAGAAGATGAACGGTGTACAGACCA	168BPLock
29[84]	GGGTAATTGAGCGCTAATATCAGAGAGATAACCCACAAGAAT	168BPLock
29[42]	ATTAGACGGGAGAATTAACTGGGCGCATAGGCTGGCTGACCT	168BPLock
29[21]	AACATAAAAACAGGGAAGCGCTCATCAAGAGTAATCTTGACA	168BPLock
28[188]	GATAAATTGTGTCGAAATCCGAATAGCTATCTTACCGAAGCC	168BPLock
28[167]	CGACCTGCTCCATGTTACTTAAGCAAGAAACAATGAAATAGC	168BPLock
28[146]	GCCGGAACGAGGCGCAGACGGTGAGTTAAGCCCAATAATAAG	168BPLock
28[125]	TCAATCATAAGGGAACCGAACTGACCAACTTTGAAAGAGGAC	168BPLock

15[245]	TGGCTTAGCGTTTTTTTATCACAAAAT	CapturingStrand
0[244]	GCCGCCATTTTGCTAGTTAGCAGAACTG	CapturingStrand

26[41]	GTGTTGTTCCAGTTTGGAACAAGAGTCCACTATTAAAGAACG	ClosedLock
25[357]	TCCAGAACAATATTACCGCCAGCCATTGCAACAGGAAAAACG	ClosedLock
27[357]	TAGCTTAGATTAAGACGCTGAGAAGAGTCAATAGTGAATTTA	ClosedLock
24[41]	GACAGTGCGGCCCTGCCATCTGTAAGCAACTCGTCGGTGGGC	ClosedLock
27[196]	GGTCAGGATTAGAGAGTACCTGACCGGAAGCAAACTCCAACA	ClosedLock
25[196]	CCGATTAAAGGGATTTTAGACGAGCGGGAGCTAAACAGGAGG	ClosedLock
24[202]	ACAAATAAATCCTCATTAAAGGATTGGCCTTGATATTCACAA	ClosedLock
26[202]	TCGGTTTATCAGCTTGCTTTCCAAAAGGAGCCTTTAATTGTA	ClosedLock

26[223]	CAAAAGGAGCCTTTAATTGTATCGGTTTATCAGCTTGCTTTC	OpenLock
24[223]	GATTGGCCTTGATATTCACAAACAAATAAATCCTCATTAAAG	OpenLock
25[175]	GAGCGGGAGCTAAACAGGAGGCCGATTAAAGGGATTTTAGAC	OpenLock
27[175]	GACCGGAAGCAAACTCCAACAGGTCAGGATTAGAGAGTACCT	OpenLock
24[20]	GTAAGCAACTCGTCGGTGGGCGACAGTGCGGCCCTGCCATCT	OpenLock
27[378]	GAAGAGTCAATAGTGAATTTATAGCTTAGATTAAGACGCTGA	OpenLock
25[378]	GCCATTGCAACAGGAAAAACGTCCAGAACAATATTACCGCCA	OpenLock
26[20]	AGAGTCCACTATTAAAGAACGGTGTTGTTCCAGTTTGGAACA	OpenLock

9[245]	TACTGTGACCGATTGCATGATAGGTGGCTACCAGC	EGFP-Nter12base
15[245]	TGGCTTAGCGTTTTATCACAAAATTTAACAATTAAGAG	EGFP-Cter12base
9[245]	TTGTACTGTGACCGATTGCATGATAGGTGGCTACCAGC	EGFP-Nter15base
15[245]	TGGCTTAGCGTTTTATCACAAAATTTAACAATTAAGAGCTG	EGFP-Cter15base
9[245]	CGGTTGTACTGTGACCGATTGCATGATAGGTGGCTACCAGC	EGFP-Nter18base
15[245]	TGGCTTAGCGTTTTATCACAAAATTTAACAATTAAGAGCTGGGA	EGFP-Cter18base
0[244]	GCCGCCATTTTGCTAGTTAGCAGAACTGTTTCGGTCACAGTACAACCG	FAM
15[245]	TGGCTTAGCGTTTTATCACAAAATTTGGTTGTACTGTGACCGATTC	BHQ1
1[208]	CCAGAATGGAAAGCGCAGTAAAGTATTAAGAGGCTGAGTACAGGGGTGTGGGGACAGGGGTGTGGGGTACATCAGAGCCACCACCGGATCAAAATCACCGGAA	G-quadruplex
5[208]	AGGTGAATTTCTTAAACAACCAGGCAAAGCGCCATTCTACAGGGGTGTGGGGACAGGGGTGTGGGGTACATGACGTTAGTAAATGAATTTTGTCGTCTTTCCA	G-quadruplex
7[208]	TCAGGAACGAGGGTAGCAACGGCTACAGATACAGGGGTGTGGGGACAGGGGTGTGGGGTACATTTTATTTTGTCACAATGACACCACGGAATAAG	G-quadruplex
13[208]	ACGAGTAGATTTAGTTATTCCCAATTCTGCGATACAGGGGTGTGGGGACAGGGGTGTGGGGTACATAGGGTGAGAAAGGCCGGAGACAGTCAGAGT	G-quadruplex
17[208]	AAATCAAGATTAGTTGGAGGTTTTGAAGCCTTTACAGGGGTGTGGGGACAGGGGTGTGGGGTACATAATCCCCCTCAAATGCTTTCGAGCTTCAAAGCGAACC	G-quadruplex
19[208]	ATTTGCACGTAAAACACTACCATATCAAAATTTACAGGGGTGTGGGGACAGGGGTGTGGGGTACATTTGTGAATTACCTTATGCGGCTTTCCTCGTTAGAATC	G-quadruplex
1[208]	CCAGAATGGAAAGCGCAGTAAAGTATTAAGAGGCTGAGTGCGCGCGGATCCGCGCAAGCGCGCGCCTAGGCGCGTCAGAGCCACCACCGGATCAAAATCACCG	BamHI
5[208]	AGGTGAATTTCTTAAACAACCAGGCAAAGCGCCATTCTGCGCGCGGATCCGCGCAAGCGCGCGCCTAGGCGCGTGACGTTAGTAAATGAATTTTGTCGTCTTT	BamHI
7[208]	TCAGGAACGAGGGTAGCAACGGCTACAGATGCGCGCGGATCCGCGCAAGCGCGCGCCTAGGCGCGTTTTATTTTGTCACAATGACACCACGGAATAAG	BamHI
13[208]	ACGAGTAGATTTAGTTATTCCCAATTCTGCGATGCGCGCGGATCCGCGCAAGCGCGCGCCTAGGCGCGTAGGGTGAGAAAGGCCGGAGACAGTCAGAGT	BamHI
17[208]	AAATCAAGATTAGTTGGAGGTTTTGAAGCCTTTGCGCGCGGATCCGCGCAAGCGCGCGCCTAGGCGCGTAATCCCCCTCAAATGCTTTCGAGCTTCAAAGCGA	BamHI
19[208]	ATTTGCACGTAAAACACTACCATATCAAAATTTGCGCGCGGATCCGCGCAAGCGCGCGCCTAGGCGCGTTTGTGAATTACCTTATGCGGCTTTCCTCGTTAGA	BamHI
1[208]	CCAGAATGGAAAGCGCAGTAAAGTATTAAGAGGCTGAGTTCAGCCGCTGTCACACGCACAG	miRNA-210
1[208]	TCAGCCGCTGTCACACGCACAGTCAGAGCCACCACCGGATCAAAATCACCGGAAC	miRNA-210
5[208]	AGGTGAATTTCTTAAACAACCAGGCAAAGCGCCATTCTTCAGCCGCTGTCACACGCACAG	miRNA-210
5[208]	TCAGCCGCTGTCACACGCACAGTGACGTTAGTAAATGAATTTTGTCGTCTTTCCA	miRNA-210

7[208]	TCAGGAACGAGGGTAGCAACGGCTACAGATTCAGCCGCTGTCACACGCACAG	miRNA-210
7[208]	TCAGCCGCTGTCACACGCACAGTTTTATTTTGTCACAATGACACCACGGAATAAG	miRNA-210
13[208]	ACGAGTAGATTTAGTTATTCCCAATTCTGCGATTCAGCCGCTGTCACACGCACAG	miRNA-210
13[208]	TCAGCCGCTGTCACACGCACAGTAGGGTGAGAAAGGCCGGAGACAGTCAGAGT	miRNA-210
17[208]	AAATCAAGATTAGTTGGAGGTTTTGAAGCCTTTTCAGCCGCTGTCACACGCACAG	miRNA-210
17[208]	TCAGCCGCTGTCACACGCACAGTAATCCCCCTCAAATGCTTTCGAGCTTCAAAGCGAACCA	miRNA-210
19[208]	ATTTGCACGTAAAACACTACCATATCAAAATTTTCAGCCGCTGTCACACGCACAG	miRNA-210
19[208]	TCAGCCGCTGTCACACGCACAGTTTGTGAATTACCTTATGCGGCTTTCCTCGTTAGAATCA	miRNA-210
